# TSPAN18 facilitates bone metastasis of prostate cancer by protecting STIM1 from TRIM32-mediated ubiquitination

**DOI:** 10.1186/s13046-023-02764-4

**Published:** 2023-08-05

**Authors:** Qianghua Zhou, Xu Chen, Kai Yao, Yangjie Zhang, Haixia He, Hao Huang, Hao Chen, Shengmeng Peng, Ming Huang, Liang Cheng, Qiang Zhang, Ruihui Xie, Kaiwen Li, Tianxin Lin, Hai Huang

**Affiliations:** 1 Department of Urology, Sun Yat-sen Memorial Hospital, Sun Yat-sen University, 107th yanjiangxi road, Guangzhou, 510120 China; 2 Guangdong Provincial Key Laboratory of Malignant Tumor Epigenetics and Gene Regulation, Sun Yat-Sen Memorial Hospital, Sun Yat-Sen University, Guangzhou, 510120 China; 3 Department of urology, Sun Yat-sen University Cancer Center, Guangzhou, 510060 China; 4 Department of Radiation Oncology, Sun Yat-sen Memorial Hospital, Sun Yat-sen University, Guangzhou, 510120 China; 5 Guangdong Provincial Clinical Research Center for Urological Diseases, Guangzhou, 510120 Guangdong China; 6 Department of Urology, The Sixth Affiliated Hospital of Guangzhou Medical University, Qingyuan People’s Hospital, Qingyuan, 511518 Guangdong China

**Keywords:** Prostate cancer, Bone metastasis, STIM1, TSPAN18, TRIM32

## Abstract

**Background:**

Bone metastasis is a principal cause of mortality in patients with prostate cancer (PCa). Increasing evidence indicates that high expression of stromal interaction molecule 1 (STIM1)-mediated store-operated calcium entry (SOCE) significantly activates the calcium (Ca^2+^) signaling pathway and is involved in multiple steps of bone metastasis in PCa. However, the regulatory mechanism and target therapy of STIM1 is poorly defined.

**Methods:**

Liquid chromatography-mass spectrometry analysis was performed to identify tetraspanin 18 (TSPAN18) as a binding protein of STIM1. Co-IP assay was carried out to explore the mechanism by which TSPAN18 inhibits STIM1 degradation. The biological function of TSPAN18 in bone metastasis of PCa was further investigated in vitro and in vivo models.

**Result:**

We identified that STIM1 directly interacted with TSPAN18, and TSPAN18 competitively inhibited E3 ligase tripartite motif containing 32 (TRIM32)-mediated STIM1 ubiquitination and degradation, leading to increasing STIM1 protein stability. Furthermore, TSPAN18 significantly stimulated Ca^2+^ influx in an STIM1-dependent manner, and then markedly accelerated PCa cells migration and invasion in vitro and bone metastasis in vivo. Clinically, overexpression of TSPAN18 was positively associated with STIM1 protein expression, bone metastasis and poor prognosis in PCa.

**Conclusion:**

Taken together, this work discovers a novel STIM1 regulative mechanism that TSPAN18 protects STIM1 from TRIM32-mediated ubiquitination, and enhances bone metastasis of PCa by activating the STIM1-Ca^2+^ signaling axis, suggesting that TSPAN18 may be an attractive therapeutic target for blocking bone metastasis in PCa.

**Supplementary Information:**

The online version contains supplementary material available at 10.1186/s13046-023-02764-4.

## Background

Prostate cancer (PCa) is the second most frequently diagnosed malignancy in men and the fifth leading cause of cancer-associated death in men, attributed mainly to incurable bone metastasis [1]. The 5-year survival of PCa patients experiencing bone metastasis or skeletal-related events is nearly 70% lower than that of PCa patients without bone metastasis (30% vs. 100%) [2]. Even worse, the current recommended treatment options fail to markedly improve the prognosis of patients with bone- metastatic PCa [3–6]. Thus, there is a desperate need to identify the regulators of bone metastasis in PCa.

Bone metastasis of PCa is a complex process that consists of multiple stages, including a decrease in cell adhesion, followed by invasion, intravasation, circulation, extravasation and colonization in bone [7]. The calcium (Ca^2+^) signaling pathway has been reported to participate in several stages of bone metastasis [8]. For instance, elevated intracellular Ca^2+^ levels can promote epithelial-mesenchymal transition (EMT) by upregulating zinc finger E-box-binding homeobox 1 (ZEB1) expression in PCa cells [9]. Ca^2+^ entry can also facilitate the migration and invasion of PCa cells through the phosphatidylinositol 3-kinase (PI3K) signaling pathway [10]. Notably, elevated Ca^2+^ promotes bone resorption via activation of the peptide parathyroid hormone-related protein (PTHrP)/receptor activator of nuclear factor-Κb (RANK) axis, facilitating bone colonization of PCa cells [11–13]. Unlike that of other secondary messengers, homeostasis of Ca^2+^ within cells is tightly regulated by multiple pathways related to Ca^2+^ influx and efflux.

Stromal interaction molecule 1(STIM1)/Orai1-mediated store-operated Ca^2+^ entry (SOCE) is a prominent route of Ca^2+^ entry in nonexcitable cells, including PCa cells [14, 15]. Normally, upon receiving the stimulus of Ca^2+^ depletion within the endoplasmic reticulum (ER), STIM1 translocates to ER-plasma membrane (PM) junctions and interacts with Orai1, which is widely distributed in PM, and subsequently augments Ca^2+^ influx through Orai1 channels [16]. It has been reported that upregulated SITM1 expression can promote processes related to tumor progression, including metastasis, via aberrant activation of Ca^2+^-dependent signaling pathways and is associated with shortened patient survival across multiple cancer types [17–21]. Although the oncogenic role of STIM1 has been well elucidated, the underlying regulatory mode of STIM1 remains largely unclear.

Tetraspanins (TSPANs) are small transmembrane glycoproteins containing four highly conserved membrane-spanning domains, expressed in all multiple organisms. Generally, TSPANs are known to regulate subcellular localization, lateral mobility and clustering at the cell surface of their interactive ‘partner proteins’ including cell adhesion proteins, cell surface receptors, metalloproteinases, and intercellular signaling molecules, and thereby engage in a plethora of cellular functions [22, 23]. Previous studies demonstrated that TSPAN18 could maintain stability of homophilic adhesion molecule cadherin 6B in chick embryos and regulate endothelial cell Orai1/Ca^2+^ signaling and von Willebrand factor release in response to inflammatory stimuli [24, 25]. However, the function of TSPAN18 in pathological processes, particularly in tumourigenesis and metastasis is unknown.

In this study, we first revealed that TSPAN18 directly interacts with STIM1 and stabilizes it by blocking its tripartite motif containing 32 (TRIM32)-mediated ubiquitination. Functionally, we demonstrated that TSPAN18 promotes PCa cells metastasis by modulating the STIM1-dependent Ca^2+^ signaling pathway. Moreover, xenograft tumor models and clinical specimens further identified the oncogenic role of TSPAN18 mediated by its positive regulation of STIM1. Overall, our data indicated that TSPAN18 facilitates bone metastasis by activating the STIM1-Ca^2+^ signaling axis in PCa.

## Methods

### Cell culture and reagents

Human normal prostate cell WPMY-1, and PCa cell lines DU145, PC-3, C4-2 and LnCAP, and vascular endothelial cell HUEVC, and HEK-293T cells were obtained from the American Type Culture Collection (ATCC). Cells were cultured with RPMI-1640 or DMEM(Gibco) supplemented with FBS (10%, Gibco), penicillin (100 U/mL, Gibco), and streptomycin (100 μg/mL, Gibco). All cells tested negative for mycoplasma contamination. Puromycin, G418, SKF96365, CHX, MG132 and Baf were purchased from Selleck Chemicals.

### Antibodies and plasmids

All antibodies used in the present study were listed in Supplementary table 1. The Flag-TSPAN18, Myc-STIM1, Myc-STIM1 CC1, Myc-STIM1 CAD, Myc-STIM1 CCA-CAD, Myc-STIM1 CT, His-TRIM32, and HA-ubiquitin plasmids were sub-generated by inserting the corresponding full-length cDNA into the 3xFlag, Myc, His or HA-pcDNA 3.1 vector. TSPAN18 or scramble shRNA was inserted into the pLKO.1-puro vector. Full-length TSPAN18-Flag or Luc was inserted into the pCDH-puro vector to generate stable cell lines. The GST-TSPAN18 and GST-vector plasmids were constructed with the pGEX-4T-2 vector.

### Lentivirus packaging and infection

This method was performed according to a previous study [26]. In brief, lentiviral packaging was accomplished in HEK-293T cells transfected with the psPAX2 and pMD2.G plasmids, along with the shRNA-TSPAN18 pLKO.1-puro, TSPAN18-pCDH-puro, or corresponding control plasmid. DU145 and PC-3 cells were infected with the above-described virus particles, followed screening with 10 μg/mL Polybrene. In addition, stable PC-3 cells were further infected with virus particles expressing Luc-G418, and were screened with G418.

### RNA isolation, qRT-PCR, and RNA sequencing

The detailed procedures of these methods were described in our previous study [27]. The expression of all genes was normalized to that of GAPDH. The primer sequences used to amplify all targeted genes are listed in Supplemental table 2. Following RNA extraction, Library construction and sequencing were finished by Annoroad Gene Technology (Beijing, China), in line with previous study [27]. The raw RNA-sequencing data have been uploaded to Gene Expression Omnibus (GSE 217342).

### Western blot (WB) assay

WB assay were conducted as described in our previous study [27]. GAPDH serves as a loading control. At least three independent biological replicates were conducted.

### Wound healing and transwell assays

These assays were performed to evaluate the migration and invasion of PCa cells *in vitro*, and the details were presented in a previous study [28]. The experiments were repeated thrice.

### Immunofluorescence (IF) staining

Following separate treatments, cells were fixed with paraformaldehyde, blocked with BSA, and incubated with primary antibodies overnight at 4 ℃. The next day, the cells were incubated with fluorophore-conjugated secondary antibodies (Life Technologies) for 1h at RT and counterstained with DAPI (Life Technologies) for 10 min. Fluorescence images were acquired using confocal laser-scanning microscopy at 200 × and 400×microscope (Olympus BX61, Tokyo, Japan).

### Calcium assay

The cytosolic Ca^2+^ level was measured with Fluo-4 AM (Beyotime, S1060) according to the manufacturer’s procedure. Briefly, PCa cells were incubated with 2 μM Fluo-4 for 20 min and washed with Ca^2+^-free Hank's Balanced Salt Solution 3 times. Then, confocal laser scanning microscopy at a 488 nm excitation wavelength and a 520 nm emission wavelength was performed to measure the cytosolic Ca^2+^ level.

### Immunoprecipitation (IP) assay and mass spectrometry analysis

For the IP assay, whole-cell lysates from harvested cells were incubated with the indicated primary antibodies and IgG at 4 ℃ overnight. Then, after washing with lysis buffer three times, magnetic beads were added to the cell lysates for 1 h at RT, prior to three additional washed, and the protein complexes were boiled and subjected to WB. For mass spectrometry analysis, similar to the IP assay, after washing with elution buffer, the immobilized immune complexes were used to perform proteomics screening by mass spectrometry on a MALDI-TOF MS instrument (Bruker Daltonics).

### GST pulldown assay

The GST pulldown assay was performed in line with a previous study [29]. In brief, the GST-vector, GST-TSPAN18 and His-STIM1 plasmid was transfected into *E.coli* BL21 and then induced by IPTG (Sangon, B541007). The GST-vector, GST-TSPAN18 and His-STIM1 fusion proteins were then purified with Protein Purification Kit (Beyotime, P2262 and P2229s), respectively. Then, the His-STIM1 fusion protein was co-incubated with GST-vector or GST-TSPAN18 fusion protein at 4 ℃ overnight. After that, the immunoprecipitated complexes were enriched by GST beads (GE Healthcare) and subjected to SDS–PAGE.

### Tumor metastasis assay

All animal studies performed herein were approved by the Institutional Animal Care and Use Committee of Sun Yat-sen University under approval number is 19070B. Three- to four-weeks-old male BALB/c nude mice were obtained from Shanghai SLAC Laboratory Animal Co., Ltd. and housed in groups of one to five per cage, with a standard 12-h light-dark cycle in a room maintained at 24 ℃ with free access to water and chow inside a barrier facility. A total of 2.5 × 10^6^ of the indicated PC-3 cells per mouse were injected into the caudal artery, in line with our previous study [30]. Bone metastasis was monitored and imaged weekly with a bioluminescence imaging system after tumor cell injection for six weeks. After the sacrifice of mice, limbs were scanned using a micro-CT scanner (Bruker, skyscan1276). Then, the 3D images were conducted using CTvox software (Version 2,0, Bruker) and the bone volume/total volume (BV/TV) of the region of interest (ROI) was analyzed using the CT-Analyser program [31]. After micro-CT scanning, the metastatic tumors were excised and embedded in paraffin, and the tumor tissues slides were stained with H&E to visualize the structure.

### Immunohistochemistry (IHC) and scoring

The detailed procedures for IHC in PCa tissues were described in our previous study [32]. The TSPAN18 and SITM1 immunostaining in slides were semi-quantified using the H-score on account of the staining intensity and extent. Detailly, the staining intensity was categorized in four levels as follow: 0(negative), 1(weak), 2(moderate), and 3(strong). The final staining score (0-300) was the sum of the staining extent and intensity scores (score=staining intensity × staining extent). The cutoff value for differentiating high and low TSPAN18 expression was 30, as determined by the X-tile program.

### Clinical samples

We collected 126 formalin-fixed, paraffin-embedded (FFPE) primary PCa specimens and 44 normal adjacent prostate tissues from Sun Yat-sen University Cancer Center (SYSUCC, termed Cohort1) between January 2005 and December 2014, and 113 primary PCa samples from Sun Yat-sen Memorial Hospital (SYMH, termed Cohort 2) between January 2009 and December 2013. The detailed clinicopathological features of the patients in this study were listed Supplementary table 3 [33]. Overall survival

(OS) was calculated from prostatectomy to death or the last follow-up, which was December 31, 2019. Cancer-specific survival (CSS) was defined as the time from prostatectomy to death from PCa or the last follow-up.

### Statistical analysis

All statistical analyses involved in the present study were accomplished by using SPSS version 24.0 or GraphPad Prism 8.0 software. Two-tailed Student’s *t* test was carried out to compare differences between two groups, while one-way ANOVA with Tukey’s post hoc test was performed when more than two groups were compared. The correlations between TSPAN18 or STIM1 expression and clinicopathological parameters were evaluated by using Pearson correlation analysis or the Chi-square test. Both OS and PFS curves were generated by Kaplan–Meier Plotter with the log-rank test. The adjusted survival hazard ratios (HR) and 95% confidence intervals (CIs) were estimated by a Cox proportional hazards model. Data are presented as the mean ± s.d. values. *p*<0.05 was regarded as statistical significance

## Results

### TSPAN18 interacts with the STIM1 protein in vivo and in vitro

**Fig. 1:**
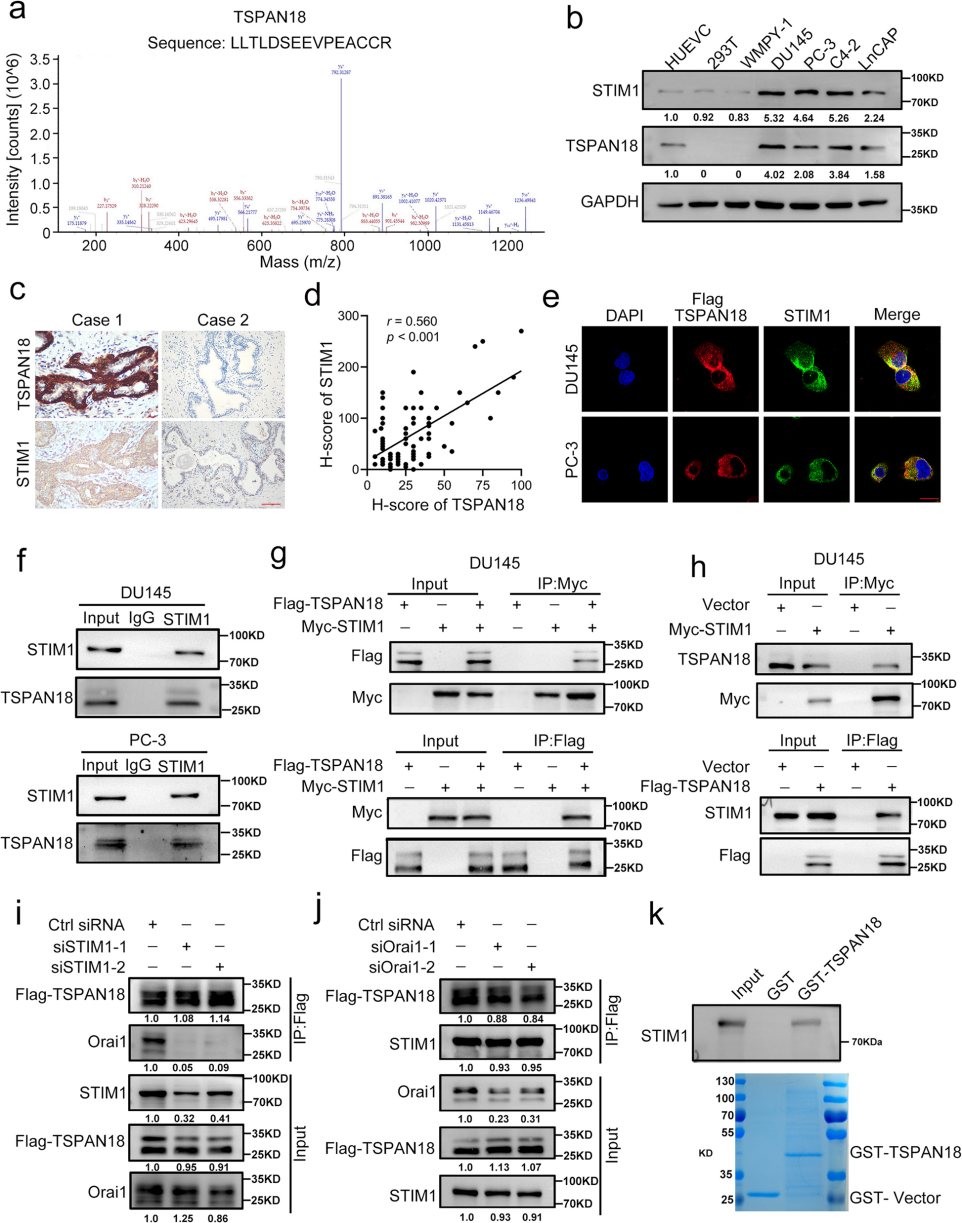
TSPAN18 interacts with STIM1 in vivo and in vitro. **a** The representative peptide of TSPAN18 from mass spectrometry. **B** Western blot analysis of the basic expression of STIM1 and TSPAN18 in one normal prostate cell line (WMPY-1) and a panel of prostate cancer cell lines. HUEVC was positive control for TSPAN18, and HEK-293T was negative control for TSPAN18. GAPDH was used as loading control. **c-d** Representative immunohistochemical staining (c) and correlation analysis (d) of TSPAN18 and STIM1 in prostate cancer. Scale bar, 100 μm. **e** Representative confocal immunostaining images for TSPAN18 (red) and STIM1 (green) in DU145 and PC-3 cells. Scale bar, 20 μm. **f** Co-immunoprecipitation (Co-IP) analysis of binding between endogenous STIM1 and endogenous TSPAN18 in DU145 (upper) and PC-3 (lower) cells using anti-STIM1 antibody. **g** Co-IP analysis of interaction between exogenous STIM1 and exogenous TSPAN18 in DU145, transfected with Flag-TSPAN18 and Myc-STIM1 plasmid using anti-Myc antibody(upper) or anti-Flag antibody (lower). **h** Co-IP analysis of binding between exogenous STIM1 and endogenous TSPAN18 (upper), or endogenous STIM1 and exogenous TSPAN18 (lower) in DU145 cells transfected with Myc-STIM1 or Flag-TSPAN18 plasmid using indicated antibodies. **i-j**. Flag-TSPAN18 overexpressing DU145 cells were transfected with siRNA targeting STIM1(i) or Orai1(j), respectively. The Co-IP analyses were conducted with anti-Flag antibody. **k** GST pulldown analysis was performed by incubating purified GST-only or GST-TSPAN18 with purified Myc-STIM1 protein, then immunoblotting analyses were performed (top panel). Coomassie blue staining of GST-TSPAN18 and GST was performed (bottom panel)

Although previous studies have demonstrated the posttranscriptional regulatory mechanism of STIM1, the posttranslational modification of STIM1 remains unclear. To investigate the underlying mechanism of STIM1 upregulation at the posttranslational level, a coimmunoprecipitation (Co-IP) assay was performed. The protein corresponding to one of the most abundant and specific bands (~30kDa) was isolated and identified as TSPAN18 by liquid chromatography-mass spectrometry (LC/MS-MS) analysis (Fig. 1a). WB analysis showed strong STIM1 and TSPAN18 expression, and positively correlated protein levels of them in a panel of PCa cell lines (Fig. 1b). Moreover, IHC staining demonstrated that STIM1 protein accumulated significantly in PCa tissues with the higher TSPAN18 expression, and statistical analysis showed that STIM1 expression was positively correlated with the abundance of TSPAN18 (r = 0.56, p < 0.001) (Fig. 1c-d). IF staining further validated that STIM1 was colocalized with TSPAN18 in the cytoplasm (Fig. 1e). To validate the interaction between STIM1 and TSPAN18, Co-IP assay were conducted and the results demonstrated that endogenous STIM1 interacted with endogenous TSPAN18 in DU145 and PC-3 cells, indicating an interaction under natural conditions (Fig. 1f). Furthermore, exogenously expressed STIM1 was detected in the Flag-TSPAN18 immunoprecipitated, and vice versa (Fig. 1g and Fig. S 1a). Moreover, endogenous STIM1 was present in the immunoprecipitated Flag-TSPAN18 complex, and vice versa (Fig. 1h). Interestingly, Co-IP assay also demonstrated that TSPAN18 could interact with Orai1 in both HEK-293T and DU145 cells, in line with previous study (Fig. S 1b-c) [25]. Considering the direct interaction between STIM1 and Orai1, we speculated that TSPAN18, STIM1 and Orai1 might form a trimolecular complex in PCa cells [34]. To further determine the binding forms among these three proteins, we knocked down STIM1 or Orai1 in Flag-TSPAN18 overexpressing DU145 cells, respectively. Then, the lysates of treated DU145 cells were immunoprecipitated with anti-Flag antibody. WB assay demonstrated that the amount of Orai1 bound to TSPAN18 was dramatically reduced in STIM1-knockdown Flag-TSPAN18 overexpressing DU145 cells, compared to control group (Fig. 1i). However, Orai1 knockdown did not weaken the interaction between STIM1 and TSPAN18, indicating that STIM1 acts as a bridging protein in the heterotrimeric complex, and both TSPAN18 and STIM1 may exert their biological functions in a Orai1-dependent manner (Fig. 1j). Moreover, a GST-pulldown assay demonstrated that highly purified recombinant GST-TSPAN18 bound to purified His-STIM1, suggesting a direct interaction (Fig. 1k). Overall, these data strongly suggested that TSPAN18 directly interacts with STIM1 in PCa cells.

### TSPAN18 increases STIM1 protein stability

**Fig. 2:**
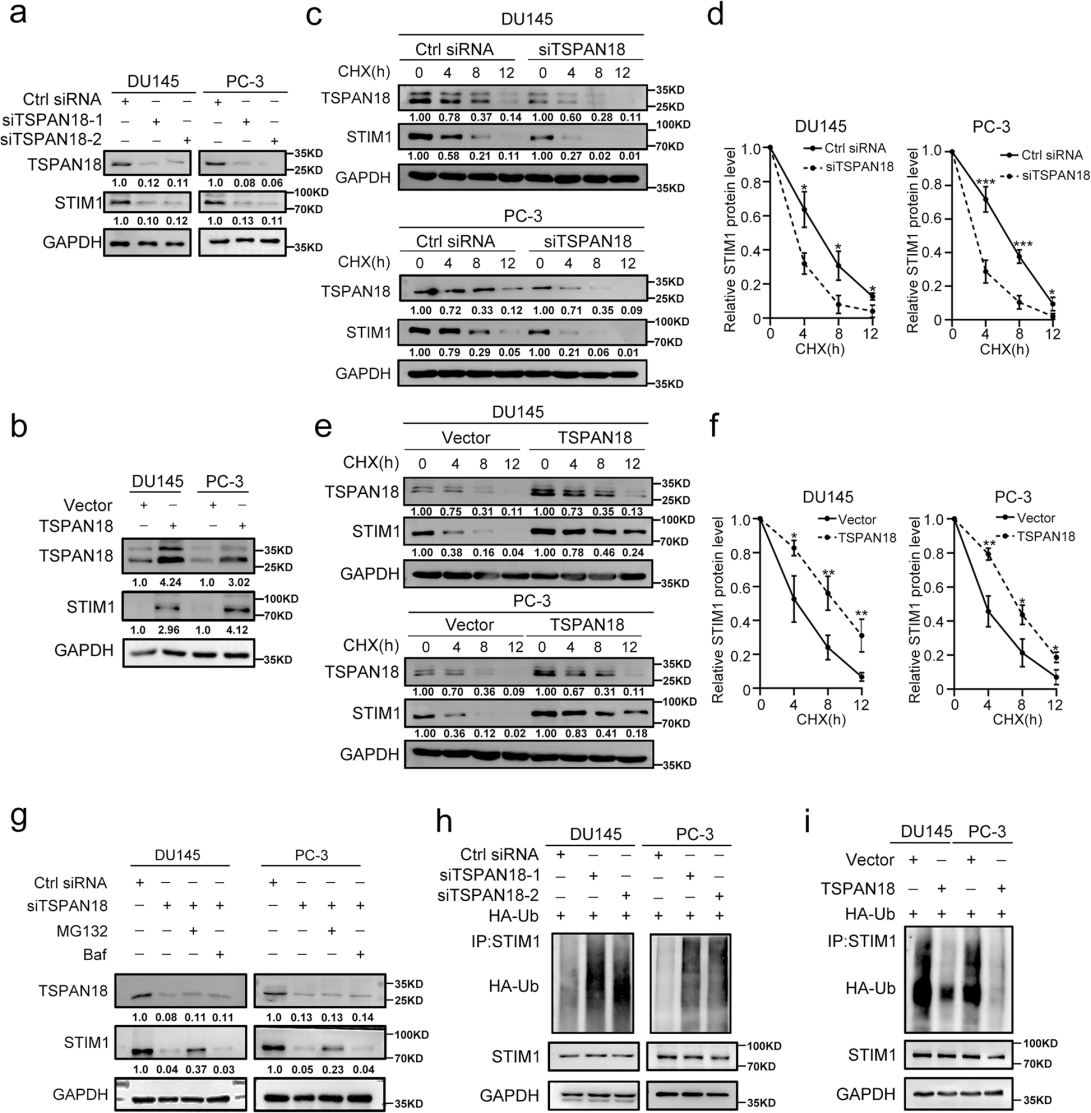
TSPAN18 maintains STIM1 stability via inhibiting ubiquitination in PCa. **a** Western blot (WB) analysis of STIM1 expression in DU145 and PC-3 cells transiently transfected with indicated siRNA. **b** WB analysis of STIM1 expression in TSPAN18-overexpressing DU145 and PC-3 cells. **c-d** The protein level of STIM1 in DU145 and PC-3 cells transfected with scramble or si-TSPAN18 were monitored by WB at indicated times after cycloheximide (CHX, 20μg/mL) (c) and were quantified by ImageJ software (d). **e-f** the protein level of STIM1 in TSPAN18 overexpressing DU145 and PC-3 cells by WB at indicated times after cycloheximide (e) and were quantified by ImageJ software (f). **g** WB analysis of lysates from DU145 or PC-3 cells transfected with scramble or si-TSPAN18 followed by treatment with DMSO, MG132 (10 μg/ml) or Baf for 4 h. Baf: Bafilomycin. **h-i** Coimmunoprecipitation (Co-IP) analysis of ubiquitination of STIM1 in TSPAN18-knockdown (h) or TSPAN18-overexpressing (i) DU145 and PC-3 cells co-transfected with Myc-STIM1 plasmid and HA-Ub plasmid. The values are expressed as the mean ± s.d. of three independent experiments. *p<0.05, **p<0.01, ***p < 0.001, Student’s *t* test

Given the close interaction of TSPAN18 and STIM1, we further examined whether TSPAN18 affects the STIM1 protein level. As shown in Fig. 2a and Fig. S 2a, WB analysis demonstrated that TSPAN18 knockdown reduced the protein level of STIM1 in PCa cells. In contrast, TSPAN18 overexpression markedly increased the STIM1 protein level (Fig. 2b and Fig. S 2b). However, neither knockdown nor overexpression of TSPAN18 changed the mRNA level of STIM1, suggesting that the potential mechanism involved posttranslational modification (Fig. S 3a-b). To validate whether TSPAN18 increases STIM1 protein stability, TSPAN18 knockdown, TSPAN18-overexpressing and the corresponding control PCa cells were treated with cycloheximide (CHX), a protein synthesis inhibitor, and the cell lysates were collected to monitor the rate of protein degradation by WB assay. As shown in Fig. 2c-f, TSPAN18 depletion decreased STIM1 expression, accompanied by a shortened half-life, however, TSPAN18 overexpression dramatically prolonged the half-life of the STIM1 protein in PCa cells, suggesting TSPAN18 as a positive regulator of STIM1 protein stability.

In general, protein degradation in eukaryotic cells is mediated mainly by the ubiquitin-proteasome system or autophagy. To determine the mechanism of STIM1 protein degradation, we treated TSPAN18 knockdown PCa cells with the proteasome inhibitor MG132 or the autophagy inhibitor bafilomycin (Baf). We found that MG132, but nor Baf, blocked STIM1 protein degradation in TSPAN18 knockdown cells (Fig. 2g and Fig. S 4). To further determine whether TSPAN18 stabilizes the STIM1 protein via de-ubiquitination, we co-transfected Myc-STIM1 and HA-Ubiquitin plasmids into TSPAN18 knockdown or TSPAN18-overexpressing PCa cells and the corresponding control PCa cells. Pulldown of STIM1 followed by WB with anti-HA (-Ub) antibody showed a marked increase in ubiquitinated STIM1 in TSPAN18 knockdown cells (Fig. 2h). Conversely, compared to that in the corresponding control cells, STIM1 polyubiquitination was dramatically decreased in TSPAN18-overexpressing PCa cells (Fig. 2i). These results suggested that TSPAN18 mainly protects STIM1 from ubiquitination-dependent proteasomal degradation.

### TSPAN18 protects STIM1 from TRIM32-mediated ubiquitination through competitive binding

**Fig. 3:**
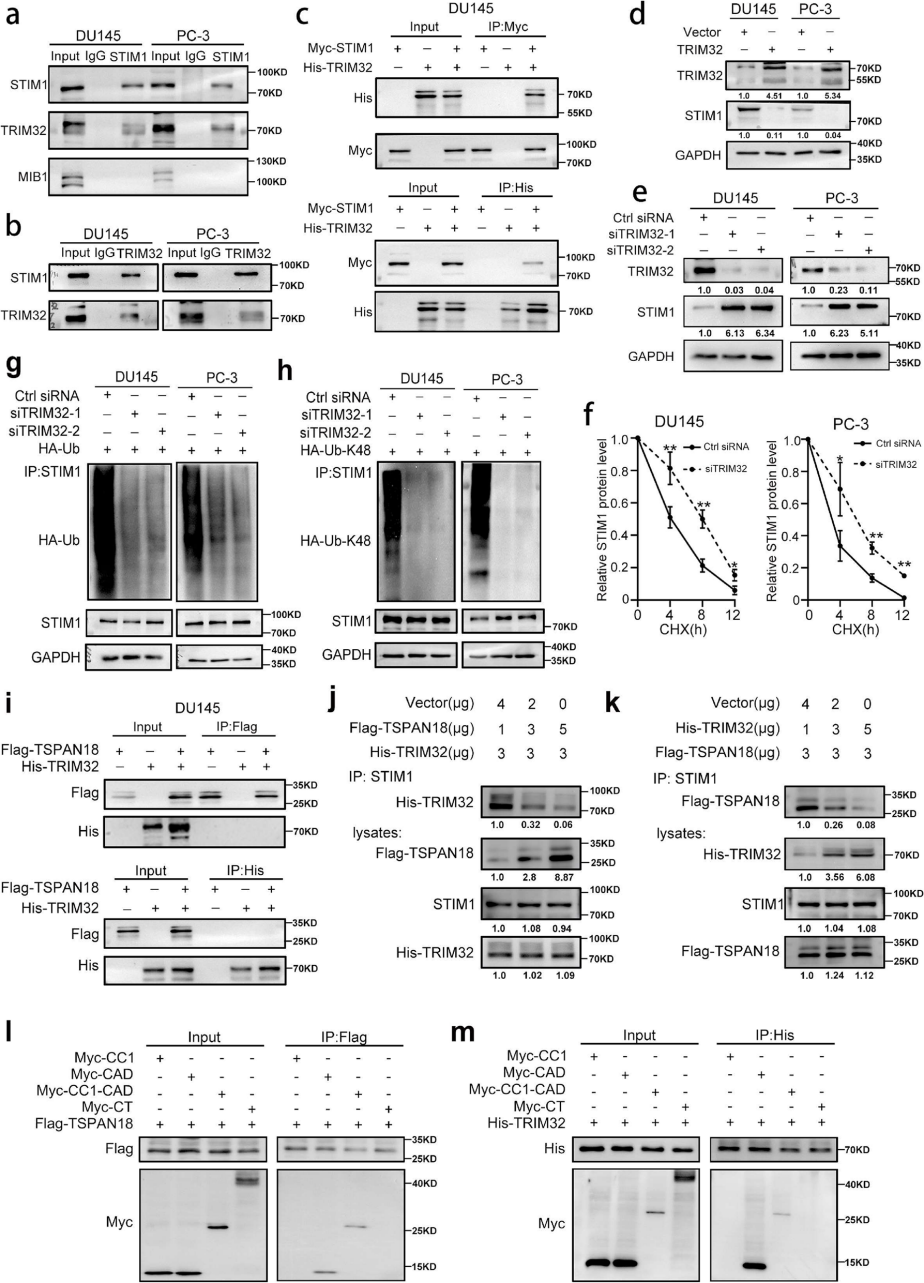
TRIM32 promotes STIM1 degradation via the ubiquitin-proteasome pathway in PCa. **a** Coimmunoprecipitation (Co-IP) analysis of interaction of STIM1 with TRIM32, MIB1 using anti-STIM1 antibody. **b** Co-IP analysis of interaction between TRIM32 and STIM1 using anti-TRIM32 antibody. **c** Co-IP analysis of interaction between exogenous STIM1 and exogenous TRIM32 in DU145 cells transfected with His-TRIM32 plasmid and Myc-STIM1 plasmid using anti-Myc antibody(up) or anti-His antibody (down). **d-e** Western blot (WB) analysis of STIM1 expression in TSPAN18-overexpressing (d) or TSPAN18-knockdown (e) DU145 and PC-3 cells. **f** the protein level of STIM1 in DU145 and PC-3 cells transfected with scramble or si-TRIM32 were monitored by WB at indicated times after cycloheximide (CHX, 20μg/mL), and were quantified by ImageJ software. The values are expressed as the mean ± s.d. of three independent experiments. *p<0.05, **p<0.01, ***p < 0.001, Student’s t test. **g-h** Co-IP analysis of ubiquitination of STIM1 in TRIM32-knockdown DU145 and PC-3 cells co-transfected with Myc-STIM1 plasmid and HA-Ub plasmid (g) or HA-Ub-K48 plasmid (h). **i** Co-IP analysis of interaction between exogenous TSPAN18 and exogenous TRIM32 in DU145 co-transfected with His-TRIM32 plasmid and Flag-TSPAN18 plasmid using anti-Flag antibody(upper) or anti-His antibody (lower). **j-k** HEK-293T cells were co-transfected with indicated plasmid for 48h, then Co-IP analysis was conducted with anti-STIM1 antibody. **l-m** WB analysis of whole-cell lysates (left) or IP (right) from cells co-expressing Myc-STIM1 CC1, Myc-STIM1 CAD, Myc-STIM1 CC1-CAD, or Myc-STIM1 CT and Flag-TSPAN18 (l) or His-TRIM32 (m) in HEK-293T cells

Generally, in the process of ubiquitination-mediated degradation, E3 ligases, which directly interact with targeted proteins, determine substrate specificity and regulate ubiquitination. To identify the candidate E3 ligases involved in TSPAN18-mediated STIM1 protein de-ubiquitination, we reanalyzed the mass spectrometry data for immunoprecipitated STIM1 and identified two candidate E3 ligases, TRIM32 and MIB1(Fig. S 5a-b). However, Co-IP assay demonstrated that only TRIM32 could interact with endogenous STIM1 within PCa cells (Fig. 3a-b). Then, we further demonstrated that exogenously expressed STIM1 was associated with TRIM32, and vice versa (Fig. 3c and Fig. S 6a). To further verify whether STIM1 is a ubiquitination substrate of TRIM32, we overexpressed TRIM32 by transient transduction and found a decrease in the endogenous STIM1 protein level (Fig. 3d and Fig. S 6b). In contrast, depletion of TRIM32 with targeted siRNAs increased the STIM1 protein level (Fig. 3e and Fig. S 6c). Moreover, TRIM32 knockdown significantly prolonged the half-life of STIM1, suggesting that TRIM32 is involved in destabilizing the STIM1 protein (Fig. 3f and Fig. S 6d).

To further explore whether TRIM32 facilitates STIM1 degradation through ubiquitination, we evaluated the polyubiquitination of STIM1 upon TRIM32 downregulation. As expected, TRIM32 knockdown dramatically decreased STIM1 polyubiquitination (Fig. 3g). Moreover, we observed that TRIM32 depletion reduced Lys48-linked ubiquitylation of STIM1 in PCa cells (Fig. 3h).

Considering the antagonistic effects of TSPAN18 and TRIM32 on the ubiquitination of STIM1, we hypothesized that TSPAN18 might disrupt the interaction between STIM1 and TRIM32, and then protect the STIM1 protein from TRIM32-mediated ubiquitination and degradation. To test this hypothesis, we first co-transfected TSPAN18-Flag and TRIM32-His into DU145 cells, but no interaction between TSPAN18 and TRIM32 was detected by Co-IP assay (Fig. 3i). Next, we co-transfected increasing concentrations of TSPAN18-Flag, along with STIM1-Myc and TRIM32-His into HEK-293T cells, and found that increasing the expression of TSPAN18 dramatically weakened the interaction between STIM1 and TRIM32 (Fig. 3j). Consistent with this finding, the TSPAN18-STIM1 interaction was also impaired by increasing TRIM32 expression (Fig. 3k). To further define the exact binding sites of TSPAN18 and TRIM32 on STIM1, we designed four cytosolic STIM1 domains according to previous study and co-transfected them with Flag-TSPAN18 or His-TRIM32 into HEK-293T cells [35]. Co-IP assay demonstrated that both TSPAN18 and TRIM32 strongly interacted with CC1-CAD and CAD domain, indicating that CAD domain is responsible for the interaction of STIM1 with TSPAN18 or TRIM32. (Fig .3l-m). Collectively, these data demonstrated that TSPAN18 stabilizes the STIM1 protein by blocking its TRIM32-mediated ubiquitination and degradation via competitive bounding.

### TSPAN18 activates the STIM1-dependent calcium signaling pathway

**Fig. 4:**
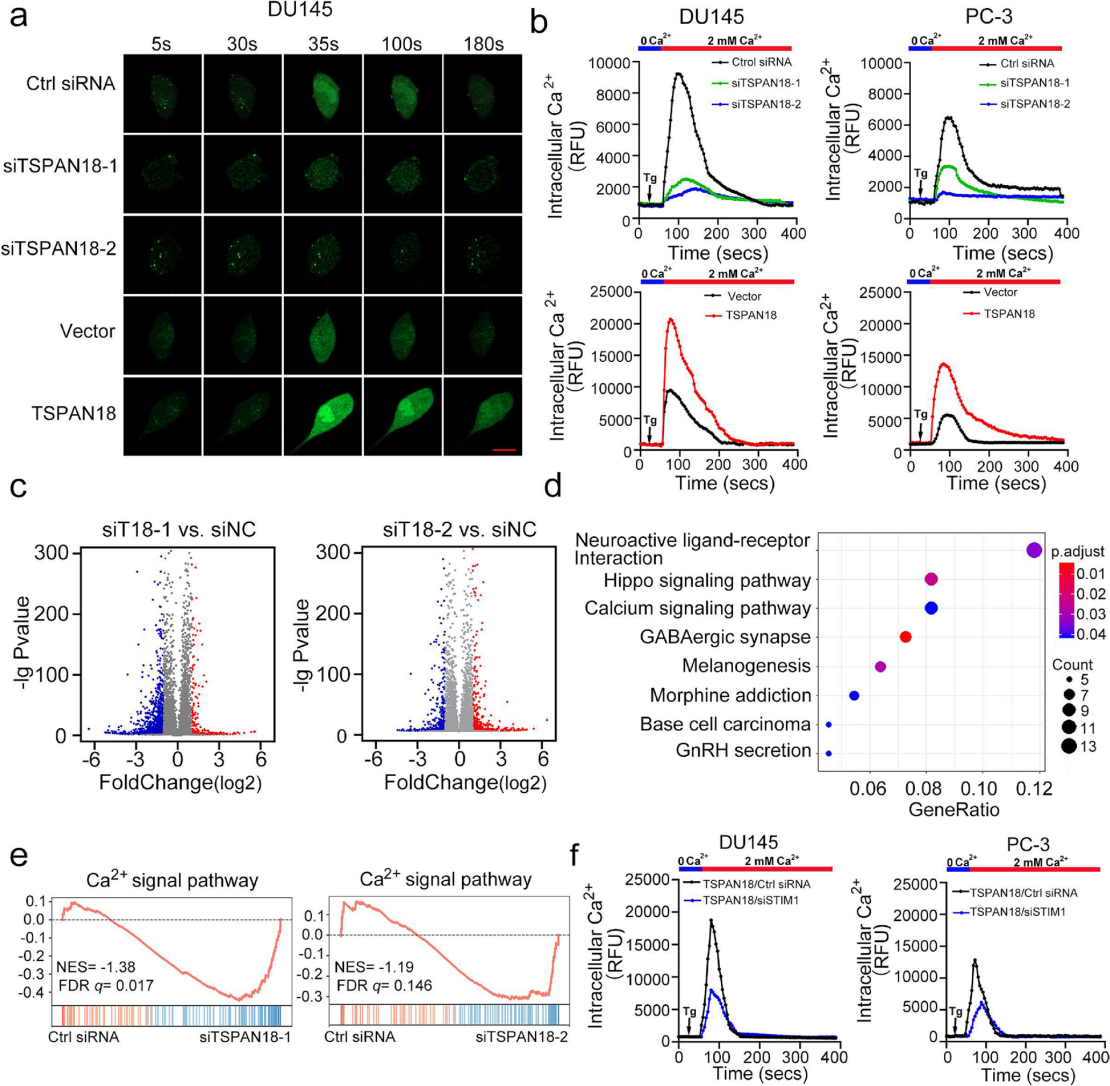
TSPAN18 activates Ca^2+^ signal pathway via STIM1-dependent manner. **a-b** After 20 minutes incubation with Ca^2+^-sensitive dye Fluo-4, the cytosolic Ca^2+^ level within indicated cells were measured by confocal laser scanning microscope. The representative time-lapse images (a) and quantification (b) of mean cytosolic Ca^2+^ level within per high-power field during measurement. Scale bar, 20 μm. RFU: Relative fluorescence unit. **c** Volcano plots displaying the genes expression change in DU145 transfected with TSPAN18-siRNAs, compared with control-siRNA. Differential expression values were plotted against *p*-value; Red dots represent the up-regulated genes and blue dots represented down-regulated genes. siT18: siTSPAN18. **d** A bubble chart exhibiting the enrichment of differentially expressed genes in signaling pathways. Size and color of the bubble represent the amount of differentially expressed genes enriched in pathways and their enrichment significance, respectively. **e** Gene set enrichment analysis (GSEA) plots of Ca^2+^ signal pathway in RNA-Seq data upon silencing of TSPAN18 in DU145 cells. NES normalized enrichment score. *p*-values in panel. **f** DU145 cells were transfected with indicated siRNAs for 48h, followed by loaded with Fluo-4, and the mean cytosolic Ca^2+^ level within per high-power field were measured by confocal laser scanning microscope

Since STIM1-activated SOCE is one of the most ubiquitous routes of evoking Ca^2+^ influx in nonexcitable cells, we speculated that TSPAN18 might facilitate Ca^2+^ influx and subsequently activate the Ca^2+^ signaling pathway in PCa cells. To confirm this hypothesis, Fluo-4 (a fluorescent Ca^2+^ indicator)-based Ca^2+^ measurement was performed. After depleting intracellular Ca^2+^ stores with thapsigargin (Tg) in the absence of extracellular Ca^2+^, Ca^2+^ influx was measured by adding 2 mM extracellular Ca^2+^. As expected, TSPAN18 knockdown markedly decreased, but TSPAN18 overexpression significantly increased Ca^2+^ influx in PCa cells compared with that in the corresponding control groups (Fig. 4a-b and Fig. S 7). Furthermore, transcriptome sequencing and gene ontology (GO) analysis revealed that the Ca^2+^ signaling pathway is one of the most enriched cancer-related pathways in TSPAN18 knockdown cells (Fig. 4c-d). GSEA further demonstrated that Ca^2+^ signaling pathway-induced genes were significantly downregulated in TSPAN18 knockdown cells (Fig. 4e). Our data further demonstrated that STIM1 knockdown strongly blocked Ca^2+^ influx in TSPAN18-overexpressing PCa cells (Fig. 4f and Fig. S 8). Together, our data indicate that TSPAN18 activates the Ca^2+^ signaling pathway in a STIM1-dependent manner.

### TSPAN18 promotes metastatic behavior of PCa cells through STIM1-calcium signal pathway

**Fig. 5:**
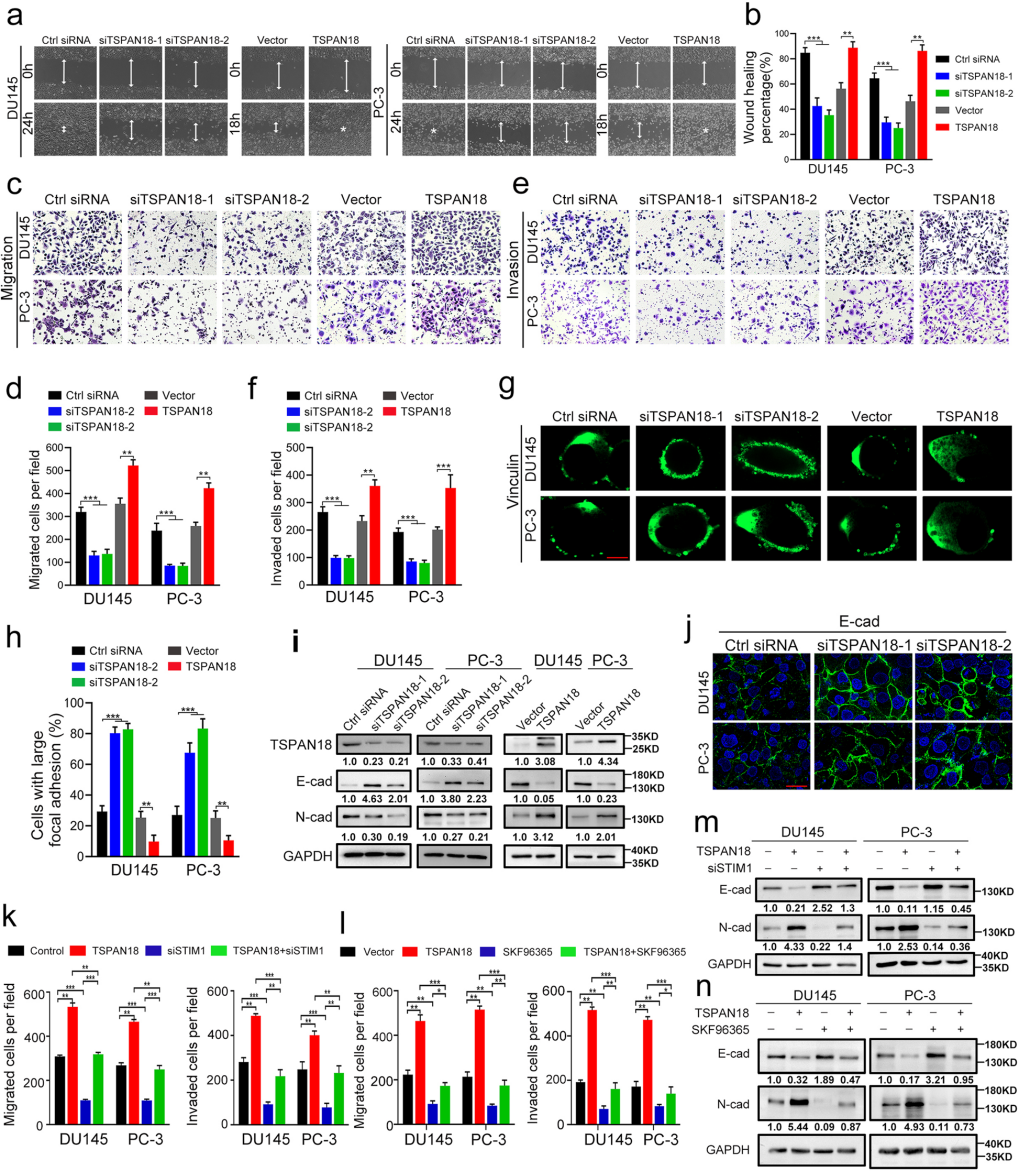
TSPAN18 promotes migration and invasion of PCa cells through STIM1-Ca^2+^ signal pathway in vitro. **a-b** Representative images (a) and histogram analysis (b) of wound-healing assay using DU145 and PC-3 cells treated as indicated. **c-d** Representative images (c) and histogram analysis (d) of migration assays using DU145 and PC-3 cells treated as indicated. **e-f** Representative images (e) and histogram analysis (f) of invasion assay using DU145 and PC-3 cells treated as indicated. **g-h** Representative images (g) and histogram analysis (h) of focal adhesions visualized by Vinculin staining within indicated cells. Scale bar, 10 μm. **i** the protein levels of N-cadherin and E-cadherin were analyzed by Western Blot (WB) within DU145 and PC-3 cells treated as indicated. **j** The immunofluorescence staining of E-cadherin within DU145 and PC-3 cells transfected with indicated siRNAs. Scale bar, 20 μm. **k** Histogram analysis of migrated (left) and invaded (right) TSPAN18-overexpressing DU145 and PC-3 cells transfected with indicated siRNAs. **l** Histogram analysis of migrated (left) and invaded (right) TSPAN18-overexpressing DU145 and PC-3 cells treated with SKF96365 or DMSO. **m** WB analysis of N-cadherin and E-cadherin within TSPAN18-overexpressing DU145 and PC-3 cells transfected with indicated siRNAs. **n** WB analysis of N-cadherin and E-cadherin within TSPAN18-overexpressing DU145 and PC-3 cells treated with SKF96365 or DMSO. The values are expressed as the mean ± s.d. of three independent experiments. **p<0.01, ***p < 0.001, Student’s* t* test or ANOVA with post hoc test

Based on the TSPAN18-mediated positive regulation of the STIM1-calcium signaling pathway, we further sought to explore the role of TSPAN18 in PCa tumorigenicity. The MTT assay and xenograft tumor mouse model demonstrated that neither knockdown nor overexpression of TSPAN18 affected proliferation of PCa cells in vivo or in vitro (Fig. S 9). However, the wound healing and Transwell assays showed that TSPAN18 knockdown dramatically decreased the migration rate and invasive capability of PCa cells. Conversely, upregulation of TSPAN18 significantly promoted the migration and invasion of PCa cells (Fig. 5a-f). Notably, TSPAN18 knockdown significantly increased, but TSPAN18 overexpression significantly reduced the size of focal adhesions and the number of large focal adhesions visualized by IF for vinculin, a major component of focal adhesions, around the periphery of the cells (Fig. 5g-h). These data indicated that TSPAN18 affected focal adhesion turnover and traction force generation in migrating cells. Additionally, a marked decrease in the expression of E-cadherin and increase in the expression of N-cadherin were observed in TSPAN18-overexpressing cells. Conversely, TSPAN18 silencing elicited E-cadherin upregulation with concomitant N-cadherin downregulation (Fig. 5i-j and Fig. S 10). the These data indicate that TSPAN18 plays a predominantly pro-metastatic role in PCa cells.

To further define whether the TSPAN18-activated STIM1-Ca^2+^ signaling pathway was responsible for the role of TSPAN18 in facilitating tumor metastasis, we transfected TSPAN18-overexpressing PCa cells and control cells with STIM1-specific siRNAs or treated them with SKF96365, an inhibitor of Ca^2+^ influx. We observed that both STIM1 knockdown and SKF96365 treatment significantly reversed the increases in the migration and invasion of TSPAN18-overexpressing PCa cells (Fig. 5k-l and Fig. S 11). Moreover, both treatments also upregulated E-cadherin expression, but downregulated N-cadherin expression in TSPAN18-overexpressing PCa cells (Fig. 5m-n and Fig. S 12). Collectively, these results suggest that TSPAN18 promotes PCa cell metastasis by activating the STIM1-dependent calcium signaling pathway.

### TSPAN18 promotes bone metastasis of PCa cells in vivo

**Fig. 6:**
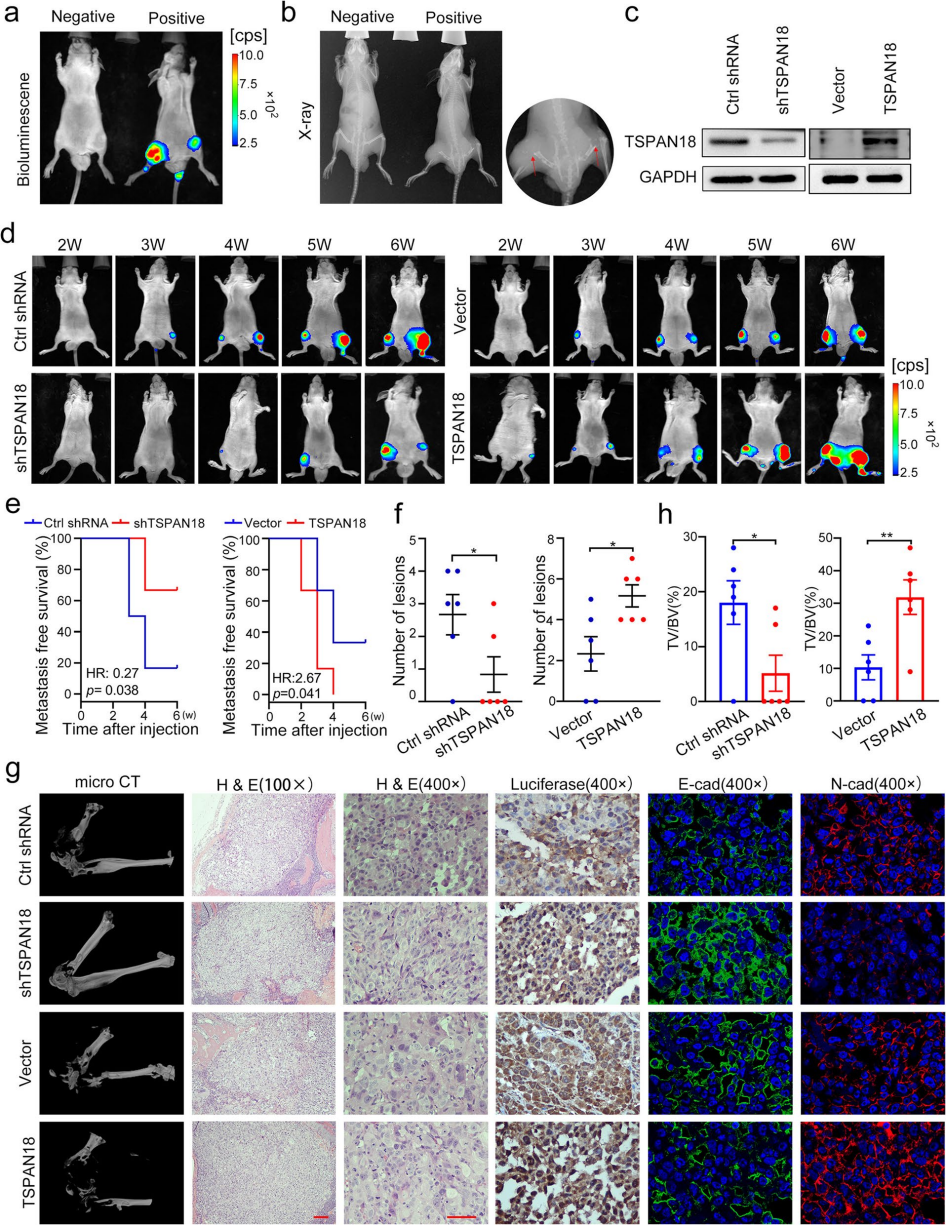
TSPAN18 promotes bone metastasis of PCa cells in vivo. **a-b** Representative images of bioluminescence (a) and X-ray (b) of bone metastasis through caudal artery injection. The red arrows show bone metastases. **c** Western blot analysis of TSPAN18 expression in stably TSPAN18-knockdown or TSPAN18-overexpressing cells and control cells. **d** TSPAN18-knockdown, TSPAN18-overexpressing and corresponding control PC-3 cells stably expressing luciferase were injected into nude mice through caudal artery, then the bone metastasis was weekly measured using an in vivo IVIS system. Representative bioluminescence images at indicated weeks from each group were shown.** e** Kaplan-Meier curves for metastasis-free survival of mice bearing PC-3 cells as indicated. **f** The counts of metastasis in indicated groups (n=6/group). **g** Representative immunohistochemical images of micro-CT, H&E and luciferase, and immunofluorescent staining of E-cadherin (green) and N-cadherin (red) in each group as indicated. The nucleus is labeled with DAPI (blue). Scale bars: red, 50 μm. **h** The tumor volume to the bone volume ratio was calculated for each mouse and presented in the plot at right. *p<0.05, **p<0.01 student’s t test

To further elucidate the potential impact of TSPAN18 on the metastatic capability of PCa cells, an experimental bone metastasis model was established in mice by injection of PCa cells via the caudal artery (Fig 6a-b). Luciferase (Luc)-expressing PC-3 cells selected for stable knockdown or overexpression of TSPAN18 by lentiviral transduction were injected via the caudal artery into nude mice (Fig. 6c). Then, bioluminescence imaging was conducted weekly to monitor the dissemination of PCa cells in real time. Strikingly, TSPAN18 knockdown significantly prolonged, but TSPAN18 overexpression markedly shortened, the time to metastasis development (Fig. 6d-e). Furthermore, TSPAN18 knockdown observably decreased, but TSPAN18 overexpression significantly increased, the number and size of metastatic foci, as determined by the luminescence intensity (Fig. 6f and Fig. S 13). Micro-CT also demonstrated that TSPAN18 knockdown significantly decreased, but TSPAN18 overexpression significantly increased, the osteolytic bone lesion area (Fig. 6g-h). In addition, both H&E staining and Luc immunostaining were performed to further verify the metastatic tumor lesions in each group (Fig. 6g). The IF assay demonstrated that N-cadherin expression was markedly decreased in TSPAN18 knockdown cell-derived bone metastatic nodules, but increased in TSPAN18-overexpressing cell-derived bone metastatic nodules. Conversely, E-cadherin expression was markedly upregulated in TSPAN18 knockdown cell-derived bone metastatic nodules but reduced in TSPAN18-overexpressing cell-derived bone metastatic nodules (Fig. 6g). Taken together, these results indicate that TSPAN18 plays an important role in facilitating PCa cell metastasis* in vivo.*

### TSPAN18 is overexpressed in human PCa tissues, and correlated with bone metastasis and poor prognosis

**Fig. 7:**
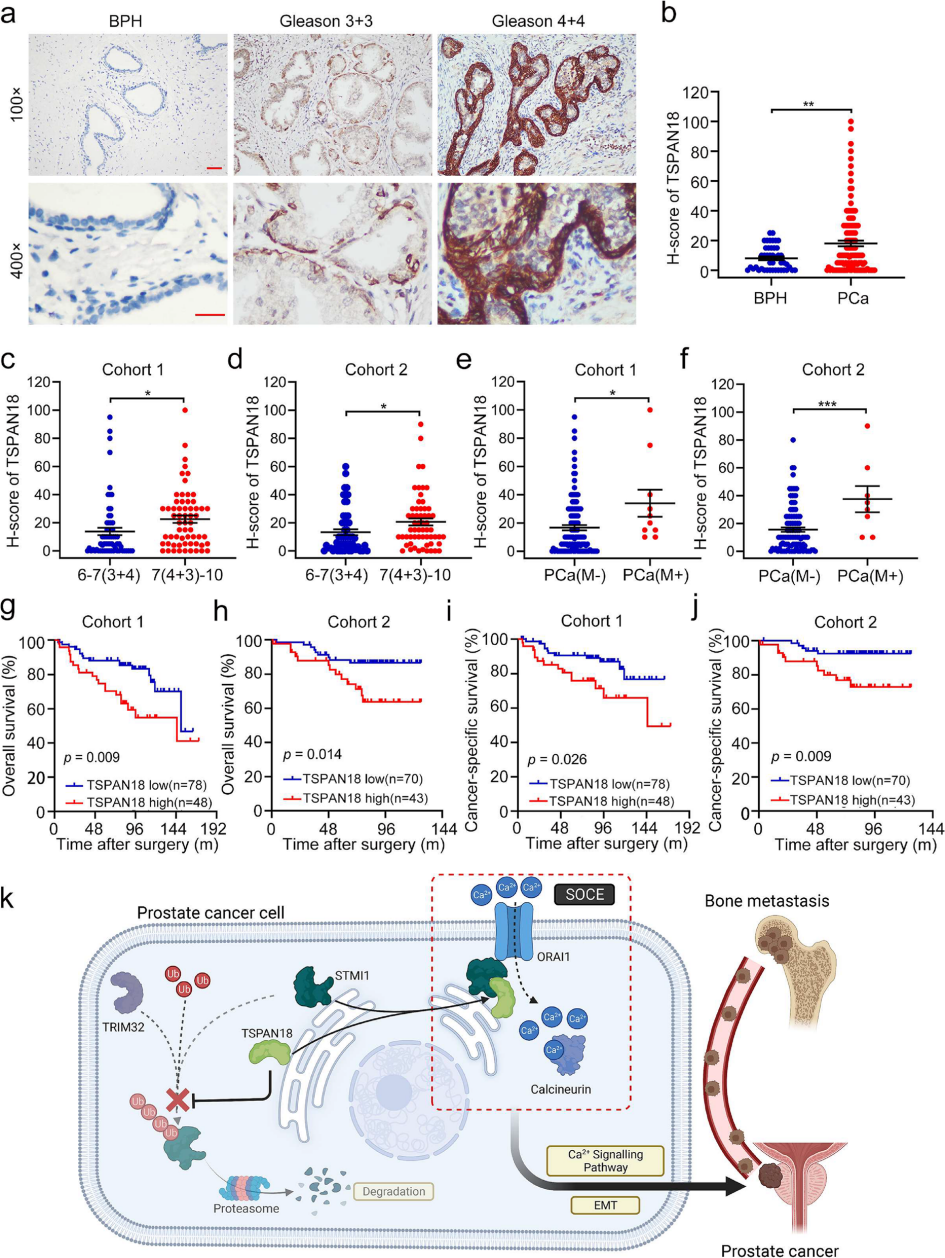
TSPAN18 overexpression correlates with bone metastasis and poor prognosis in PCa. **a** Representative immunohistochemical staining images of TSPAN18 in benign prostatic hyperplasia (BPH) and PCa with Gleason (3+3) or Gleason (4+4). Scale bars: red, 50 μm. **b** The expression difference of TSPAN18 between BPH and PCa tissues. **c-d** The expression difference of TSPAN18 between low Gleason score ((6-7(3+4)) PCa tissues and high Gleason score (7(4+3)-10) PCa tissues in Cohort 1 (c) and Cohort 2 (d). **e-f** The expression difference of TSPAN18 between PCa tissues with or without bone metastasis in Cohort 1 (e) and Cohort 2 (f). **g-h** Kaplan-Meier curves for overall survival of PCa patients with high or low expression of TSPAN18 in Cohort 1 (g) and Cohort 2 (h). **i-j** Kaplan-Meier curves for cancer-specific survial of PCa patients with high or low expression of TSPAN18 in Cohort 1 (i) and Cohort 2 (j). **k** Working model for regulation of STIM1 stability and bone metastasis of PCa by TSPAN18. The error bars mean standard deviations of three independent experiments. *p<0.05, student’s *t* test

Finally, to explore the clinical relevance of TSPAN18 expression in PCa, IHC was performed. As shown in Fig. 7a-b, TSPAN18 was highly expressed in PCa tissues compared to adjacent benign prostatic hyperplasia (BPH) tissues. Furthermore, we observed that tumor TSPAN18 expression was significantly associated with advanced Gleason score and bone metastasis, but not T stage in both cohorts, suggesting that TSPAN18 was crucial for promoting tumor metastasis (Fig. 7c-f and Fig. S 14). Next, we divided the PCa patients into two groups based on TSPAN18 expression in tumor tissue. Univariate analysis revealed that compared with patients with low TSPAN18 expression, patients with high TSPAN18 expression exhibited a more than twofold increase in the risk of mortality, especially cancer-specific deaths, in the two cohorts (OS: cohort 1, HR=2.35, 95% CI=1.21 to 4.54, *p*=0.011; cohort 2, HR=2.39, 95% CI=1.08 to 5.30, *p*=0.031; CSS: cohort 1, HR=2.77, 95% CI=1.18 to 6.59, *p*=0.019; cohort 2, HR=3.75, 95% CI=1.28 to 11.00, *p*=0.016; Supplemental table 4). Kaplan-Meier analysis also demonstrated that high TSPAN18 expression was significantly associated with decreased overall survival (OS) and cancer specific survival (CSS) in both cohorts (Fig. 7g-j). Multivariate analysis further suggested that high TSPAN18 expression in PCa was an independent risk factor for OS and CSS in cohort 1, but not in cohort 2 (Supplemental table 5). Altogether, these data indicate that TSPAN18 is highly expressed in PCa tissue and closely associated with bone metastasis and poor prognosis.

## Discussion

Bone metastasis is the main cause of PCa patient mortality; however, the underlying mechanisms are still unclear. STIM1-mediated SOCE has been found to support various cancer hallmarks, including cell viability, migration and metastasis, but the mechanism of STIM1 upregulation is not fully understood. In the present study, we demonstrated that TSPAN18 interacted with STIM1 and protected the STIM1 protein from TRIM32-mediated ubiquitination and degradation, indicating a novel mechanism of STIM1 regulation. Moreover, TSPAN18 exerted a prometastatic effect via STIM1-Ca^2+^ signaling axis, both in vivo and in vitro. Furthermore, upregulation of TSPAN18 was significantly correlated with STIM1 expression and predicted poor prognosis in PCa. These novel findings indicated that targeting the TSPAN18/STIM1 axis might be a potential therapeutic strategy for preventing bone metastasis in PCa.

Recently, accumulating studies have revealed that dysregulation of Ca^2+^ homeostasis is involved in various tumor behaviors [36–38]. As a ubiquitous pathway of evoking Ca^2+^ influx in nonexcitable cells, SOCE is involved in almost every process in the metastatic cascade. Of note, STIM1, an endoplasmic reticulum Ca^2+^ sensor, can drive tumor metastasis via the STIM1-dependent Ca^2+^ response or the modulation of focal adhesion turnover [20, 39]. One study demonstrated that microRNA-185 regulates STIM1 repression through posttranscriptional mechanism in colorectal cancer (CRC) [40]. Another study found that heat shock protein 27 maintains STIM1 protein stability in CRC [41]. However, posttranslational modification mechanism of STIM1 remains unclear. In this study, we found that STIM1 interacted with TSPAN18, a member of the transmembrane protein superfamily. Interestingly, our results demonstrated that TSPAN18 protected the STIM1 protein from proteasome-mediated degradation, but not autophagy-mediated protein degradation. Similarly, Corinne et al. demonstrated that TSPAN18 could maintain stability of homophilic adhesion molecule cadherin 6B in chick embryos [24]. Besides, one study revealed that TSPAN8 interacted with PTCH1 and maintains its stability by recruiting the deubiquitinating enzyme ATXN3 [42]. Another study demonstrated that TSPAN15 promoted the degradation of p-IκBα by ubiquitin mediated proteolysis [43]. Taken together, those findings indicate that tetraspanins function by regulating the stability, in addition to the trafficking, lateral mobility of their ‘partner proteins’, especially in cancers.

Normally, E3 ubiquitin ligases play critical roles in proteasome-mediated degradation by binding substrates to ubiquitin proteins. However, the exact E3 ubiquitin ligases involved in STIM1 protein degradation remain unclear. Here, based on the following evidences, we identified TRIM32 for the first time as an E3 ubiquitin ligase that mediates the ubiquitylation and facilitates the proteasomal degradation of STIM1. First, TRIM32 interacted with STIM1 *in vivo* and *in vitro*. Second, TRIM32 decreased the half-life of STIM1 by mediating its degradation. Third, TRIM32 enhanced the ubiquitination of STIM1, especially its Lys48-linked ubiquitylation. These data indicate that TRIM32 is a negative regulator of STIM1, contrast to TSPAN18. Thus, it is important to determine whether TSPAN18 and TRIM32 affect each other’s affinity for STIM1. Our Co-IP assays demonstrated that TSPAN18 did not interact with TRIM32; however, increasing the expression of TSPAN18 obviously inhibited the ability of TRIM32 to bind STIM1. On basis of these present findings, we speculated that the binding sites for TSPAN18 and TRIM32 in STIM1 were close to each other, which subsequently resulted in mutual exclusion. Besides, like other tetraspanins, TSPAN18 may prevent TRIM32 binding to STIM1 by regulating subcellular localization of STIM1. In addition, another possible reason it that TSPAN18 may maintain STIM1 protein stability by recruiting a deubiquitinating enzyme, like TSPAN8 does [42].

Given the critical role of STIM1 in activating the Ca^2+^ signaling pathway in cancer cells and the positive role of TSPAN18 in regulating STIM1, we tested the effect of TSPAN18 on the Ca^2+^ signaling pathway. As expected, our data demonstrated that TSPAN18 accelerated Ca^2+^ influx and subsequently activated the Ca^2+^ signaling pathway, in line with Noy et al.’s study [25]. However, the mechanisms by which TSPAN18 was indicated to regulate calcium influx in these two studies were somewhat inconsistent. Noy et al. indicated that TSPAN18 accelerates Ca^2+^ influx through interacting with Orai1 in endothelial cells. Our results revealed that TSPAN18 promoted Ca^2+^ influx by maintaining the stability of the STIM1 protein. Moreover, we demonstrated that there was a direct interaction between TSPAN18 and STIM1. Accordingly, STIM1 interacts with Orai1 and orchestrate Orai1 trafficking to form Ca^2+^ channels. Thus, we speculated that TSPAN18 directly bound to STIM1 and maintained STIM1 stability, thereby promoting Orai1 translocation and calcium channel formation, ultimately accelerating calcium influx.

Recently, the small molecule drug that target STIM1-mediated SOCE have been developed and showed an anti-tumor effect. It is reported that ML-9, a widely used inhibitor of Akt kinase, myosin light-chain kinase (MLCK) and STIM1, could effectively induce PCa cell death *in vitro*, but the vivo function is lacking [44]. Therefore, the clinical use of ML-9 remained controversial perhaps due to its intolerable side effects. Here, we found that TSPAN18 was an upstream regulator of STIM1-mediated Ca^2+^ signaling pathway, and was mainly overexpressed in tumor cells and associated with worse prognosis in PCa. Overall, targeting TSPAN18 might severed as a novel and more tumor cell-specific therapeutic target for bone metastasis of PCa.

Although our study comprehensively demonstrated that TSPAN18 can directly interact with STIM1 and protect STIM1 from E3 ligase TRIM32-mediated ubiquitination, the molecular basis of the interaction between TSPAN18 and STIM1 needs additional in-depth investigations. In particular, domain deleted mutants of both TSPAN18 and STIM1 are required to define their interacting domains. While our data shown TSPAN18 functioned mainly by activating STIM1-mediated Ca^2+^ signaling pathway, the GO analysis in present study indicated that Hippo signaling pathway might also be induced by TSPAN18. Thus, Further studies are warranted to explore whether Hippo signaling pathway also plays an essential role in TSPAN18-mediated bone metastasis of PCa.

## Conclusion

In summary, our study identified TSPAN18 as a novel caretaker of STIM1 by inhibiting its TRIM32-mediated ubiquitination and degradation, leading to metastasis *in vivo* and *in vitro*, as proposed in Fig. 7k. These data provide novel insights for exploring the reasons for abnormal activation of Ca^2+^ signaling pathway and provide a potential therapeutic target for bone metastasis of PCa.

## Supplementary Information

**Additional file 1:** **Supplemental Table 1.** The antibodies used in this study are listed as follows. **Supplemental Table 2.** The primers used in real time qPCR are listed as follows. **Supplemental Table 3.** Basic characteristics of prostate cancer patients in two cohorts. **Supplemental Table 4.** Univariate analysis of prognostic factors correlated with OS and CSS. **Supplemental Table 5.** Multivariate analysis of prognostic factors correlated with OS and CSS. **Supplemental Figure 1.** (a) Co-IPanalysis of interaction between exogenous STIM1 and exogenous TSPAN18 in HEK-293Tcells, transfected with Flag-TSPAN18 and Myc-STIM1 plasmid using anti-Mycantibody(left) or anti-Flag antibody (right). (b) Co-IP analysis of interaction between Flag-TSPAN18 and Myc-Orai1 using anti-Flag antibody (left) or anti-Mycantibody (right) in HEK-293T cells. (c)Co-IP analysis of interaction between Flag-TSPAN18 and Myc-Orai1 using anti-Flag antibody in Flag-TSPAN18 overexpressing DU145 cells. **Supplemental Figure 2.** The Western Blot analysis of TSPAN18 and STIM1 protein level in DU145 and PC-3 cells treated as indicated. The values are expressed as the mean ± s.d. of three independent experiments. **p<0.01, ***p < 0.001, ANOVA with post hoctest or Student’s t test. **Supplemental Figure 3.** The qRT-PCR analysis of TSPAN18 and STIM1 mRNA level in DU145 and PC-3 cells treatedas indicated. The values are expressed as the mean ± s.d.of three independent experiments. **p<0.01, ***p < 0.001,ANOVA with post hoc test or Student’s *t* test. **Supplemental Figure 4**. The Western Blot analysis of TSPAN18 and STIM1 protein level in DU145 and PC-3 cells treated asindicated. The values are expressed as the mean ± s.d.of three independent experiments. **p<0.01, ***p < 0.001,ANOVA with post hoc test. Baf: Bafilomycin, ns: no significance. **Supplemental Figure 5.** The representative peptide of TRIM32(a) or MIB1 (b) from mass spectrometry. **Supplemental Figure 6.** (a)Co-IP analysis of interaction between exogenous STIM1 and exogenous TRIM32 inHEK-293T cells transfected with His-TRIM32 plasmid and Myc-STIM1 plasmid usinganti-Myc antibody(left) or anti-His antibody (right). (b-c) The Western Blotanalysis of TRIM32 and STIM1 protein level in DU145 and PC-3 cells treated asindicated. The values are expressed as the mean ± s.d. of three independent experiments. ***p < 0.001, Student’s t test or ANOVA with post hoctest. (d) The protein level of STIM1 in DU145 and PC-3cells transfected with scramble or si-TRIM32 were monitored by WB at indicated times after cycloheximide (CHX, 20μg/mL). **Supplemental Figure 7.** The representative time-lapse images of cytosolic Ca^2+^level within PC-3 cells transfected with indicated siRNAs or plasmids. Scale bars: red, 50 μm. **Supplemental Figure 8.** The representative time-lapse images of cytosolic Ca^2+^level within indicated cells. Scale bars: red, 50 μm. **Supplemental Figure 9.** (a-b) Cell viability wasevaluated in TSPAN18 knockdown or overexpressing DU145 and PC-3 cells. (c)Tumor growth curves are summarized in the line chart. The average tumor volumeis expressed as the mean ± SD of six mice. (d) Representative images of thetumors of TSPAN18 knockdown or overexpression groups and their respectivecontrols. (e) Tumor weights were measured after the tumors were surgicallydissected. ns:no significance. **Supplemental Figure 10.** The Western Blot analysis of E-cadherin and N-cadherinprotein level in DU145 and PC-3 cells treated as indicated. The values are expressed as the mean ± s.d. ofthree independent experiments. **p<0.01, ***p < 0.001, ANOVA with post hoc test or Student’s* t* test. **Supplemental Figure 11.** The representative images of migration and invasion assays usingTSPAN18-overexpressing or control DU145 and PC-3 cells transfected withindicated siRNAs (a, b) or treated with SKF96365 or DMSO (c, d). **Supplemental Figure 12.** The Western Blot analysis of N-cadherin and E-cadherinprotein level in DU145 and PC-3 cells treated as indicated. The values are expressed as the mean ± s.d. ofthree independent experiments. **p<0.01, ***p < 0.001,ANOVA with post hoc test. ns: no significance. **Supplemental Figure 13.** Therepresentative bioluminescence images of the mice after 6 weeks of inoculationswith indicated PC-3 cells. **Supplemental Figure 14**. The expression difference of TSPAN18 between lowT stage PCa tissues and high T stage PCa tissues in Cohort 1 (a) and Cohort 2 (b).ns: no significance. Student’s* t* test.

## Data Availability

All the data used in the current study are available from the corresponding authors upon reasonable request.

## Abbreviations

PCa: Prostate cancer
SOCE: Store-operated calcium entry
STIM1: Stromal interaction molecule 1
TSPAN18: Tetraspanin 18
TRIM32: Tripartite motif containing 32
EMT: Epithelial-mesenchymal transition
ZEB1: Zinc finger E-box-binding homeobox 1
PI3K: Phosphatidylinositol 3-kinase
ER: Endoplasmic reticulum
Co-IP: Coimmunoprecipitation
LC/MS-MS: Liquid chromatography-mass spectrometry
IF: Immunofluorescence staining
IHC: Immunohistochemical staining
WB: Western blot assay
CHX: Cycloheximide
Baf: Bafilomycin
Tg: Thapsigargin
GO: Gene ontology
OS: Overall survival
CSS: Cancer specific survival
FFPE: Formalin-fixed, paraffin-embedded
SYSUCC: Sun Yat-sen University Cancer Center
SYSMH: Sun Yat-sen Memorial Hospital
HR: Hazard ratios
CI: Confidence intervals
