# Correction: TSPAN18 facilitates bone metastasis of prostate cancer by protecting STIM1 from TRIM32-mediated ubiquitination

**DOI:** 10.1186/s13046-025-03373-z

**Published:** 2025-04-10

**Authors:** Qianghua Zhou, Xu Chen, Kai Yao, Yangjie Zhang, Haixia He, Hao Huang, Hao Chen, Shengmeng Peng, Ming Huang, Liang Cheng, Qiang Zhang, Ruihui Xie, Kaiwen Li, Tianxin Lin, Hai Huang

**Affiliations:** 1 Department of Urology, Sun Yat-Sen Memorial Hospital, Sun Yat-Sen University, 107th yanjiangxi road, Guangzhou, 510120 China; 2 Guangdong Provincial Key Laboratory of Malignant Tumor Epigenetics and Gene Regulation, Sun Yat-Sen Memorial Hospital, Sun Yat-Sen University, Guangzhou, 510120 China; 3 Department of urology, Sun Yat-Sen University Cancer Center, Guangzhou, 510060 China; 4 Department of Radiation Oncology, Sun Yat-sen Memorial Hospital, Sun Yat-Sen University, Guangzhou, 510120 China; 5 Guangdong Provincial Clinical Research Center for Urological Diseases, Guangzhou, 510120 Guangdong China; 6 Department of Urology, The Sixth Afliated Hospital of Guangzhou Medical University, Qingyuan People’s Hospital, Qingyuan, 511518 Guangdong China

**Correction: J Exp Clin Cancer Res 42, 195 (2023)**

10.1186/s13046-023-02764-4

Following the publication of the original article [1], the authors identified errors in Fig. 6 and Figure S13, specifically:

- Figure 6c - The TSPAN18 protein band of TSPAN18 overexpression was misplaced.
- Figure 6e - The labels of X-axis were mislabeled.
- Figure S13 - The mice pictures of group “Ctrl shRNA” and “Vector” were misplaced and should be switched.

These errors were caused by unintentionally covering the correct image during figure preparation. The corrected figures are provided below.

**Incorrect Fig. 6**

**Fig. 6:**
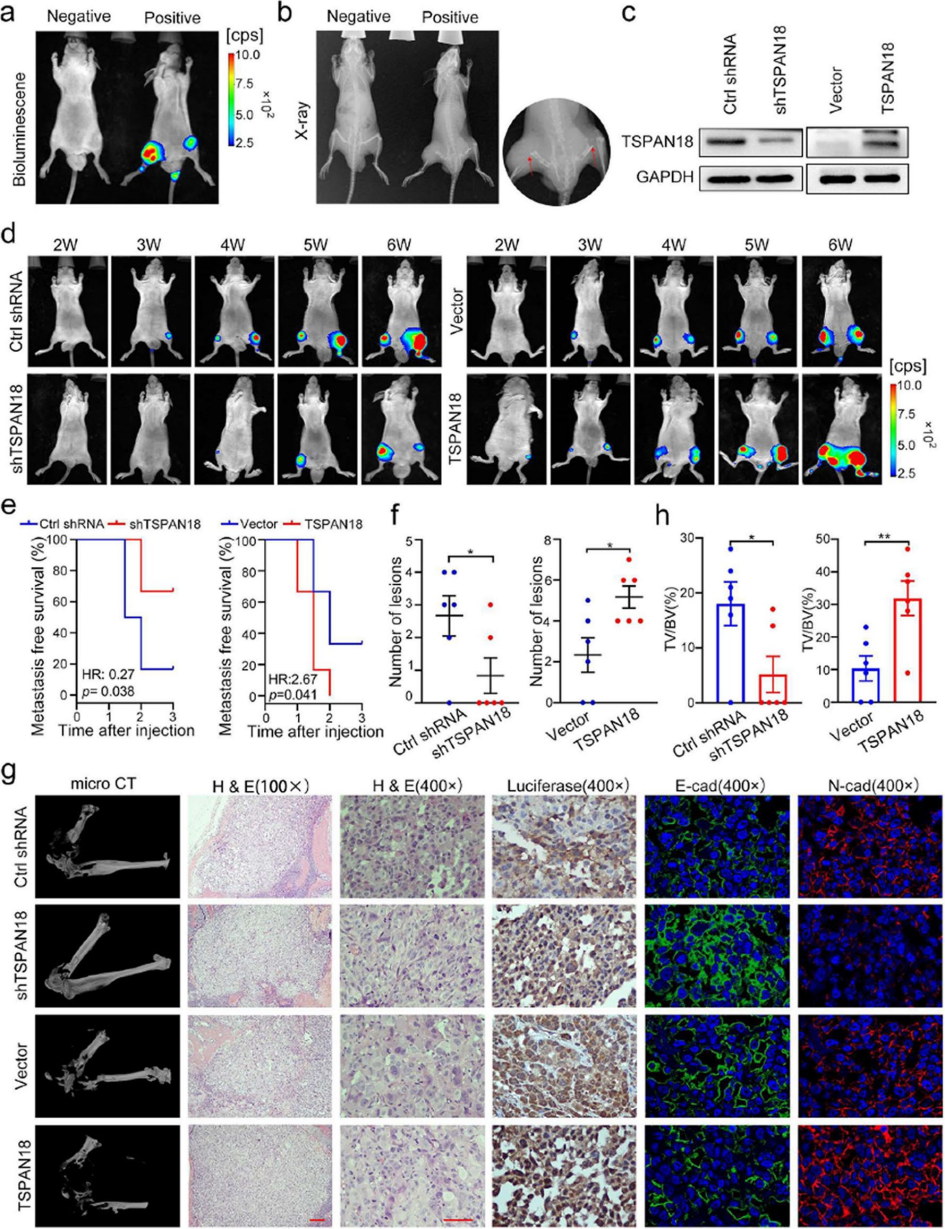
TSPAN18 promotes bone metastasis of PCa cells in vivo. **a-b** Representative images of bioluminescence (**a**) and X-ray (**b**) of bone metastasis through caudal artery injection. The red arrows show bone metastases. **c** Western blot analysis of TSPAN18 expression in stably TSPAN18-knockdown or TSPAN18-overexpressing cells and control cells. **d** TSPAN18-knockdown, TSPAN18-overexpressing and corresponding control PC-3 cells stably expressing luciferase were injected into nude mice through caudal artery, then the bone metastasis was weekly measured using an in vivo IVIS system. Representative bioluminescence images at indicated weeks from each group were shown. **e** Kaplan-Meier curves for metastasis-free survival of mice bearing PC-3 cells as indicated. **f** The counts of metastasis in indicated groups (*n* = 6/group). **g** Representative immunohistochemical images of micro-CT, H&E and luciferase, and immunofuorescent staining of E-cadherin (green) and N-cadherin (red) in each group as indicated. The nucleus is labeled with DAPI (blue). Scale bars: red, 50 μm. **h** The tumor volume to the bone volume ratio was calculated for each mouse and presented in the plot at right. *p < 0.05, **p < 0.01 student’s t test

**Correct Fig. 6**

**Fig. 6:**
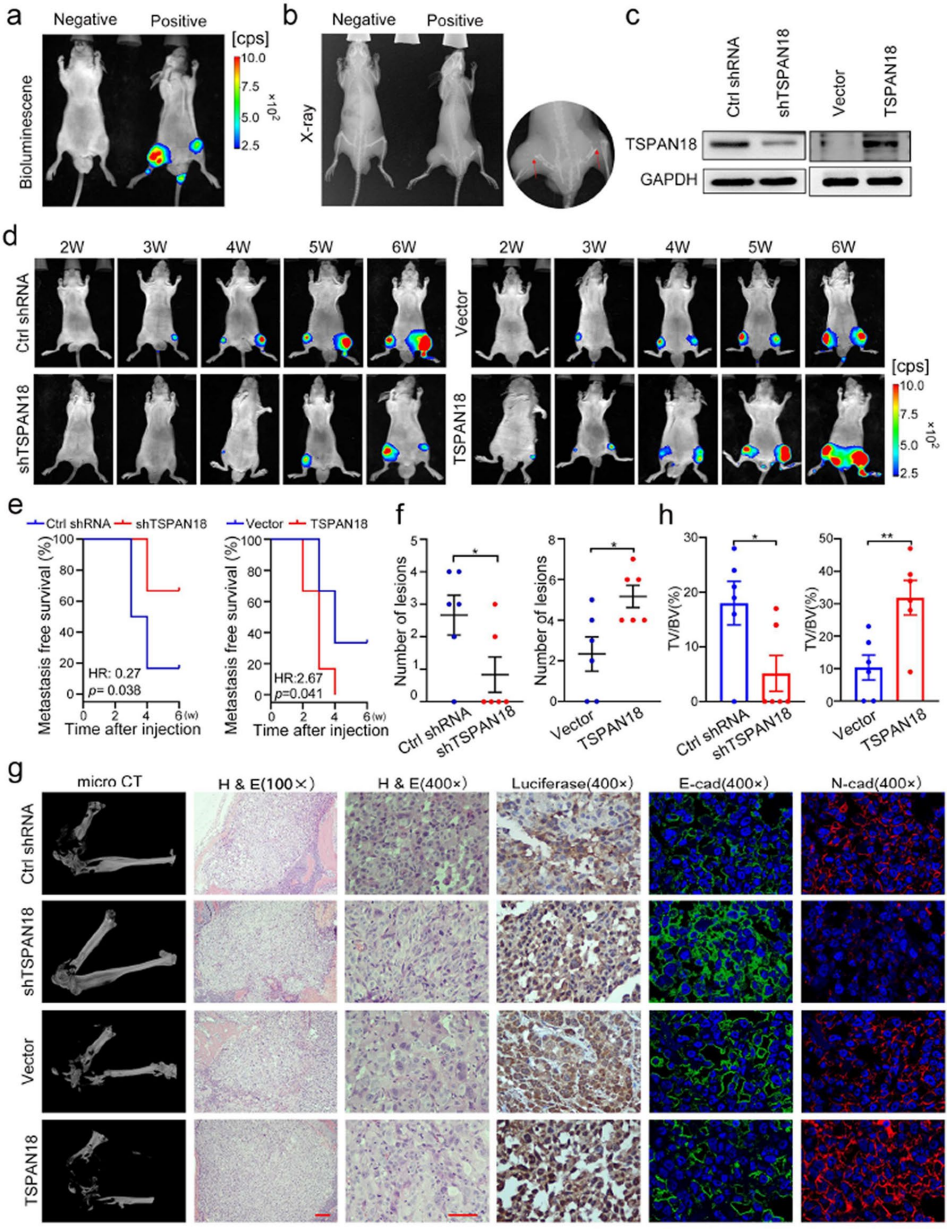
TSPAN18 promotes bone metastasis of PCa cells in vivo. **a-b** Representative images of bioluminescence (**a**) and X-ray (**b**) of bone metastasis through caudal artery injection. The red arrows show bone metastases. **c** Western blot analysis of TSPAN18 expression in stably TSPAN18-knockdown or TSPAN18-overexpressing cells and control cells. **d** TSPAN18-knockdown, TSPAN18-overexpressing and corresponding control PC-3 cells stably expressing luciferase were injected into nude mice through caudal artery, then the bone metastasis was weekly measured using an in vivo IVIS system. Representative bioluminescence images at indicated weeks from each group were shown. **e** Kaplan-Meier curves for metastasis-free survival of mice bearing PC-3 cells as indicated. **f** The counts of metastasis in indicated groups (*n* = 6/group). **g** Representative immunohistochemical images of micro-CT, H&E and luciferase, and immunofuorescent staining of E-cadherin (green) and N-cadherin (red) in each group as indicated. The nucleus is labeled with DAPI (blue). Scale bars: red, 50 μm. **h** The tumor volume to the bone volume ratio was calculated for each mouse and presented in the plot at right. *p < 0.05, **p < 0.01 student’s t test

**Incorrect Figure S13**

**Supplemental Fig. 13:**
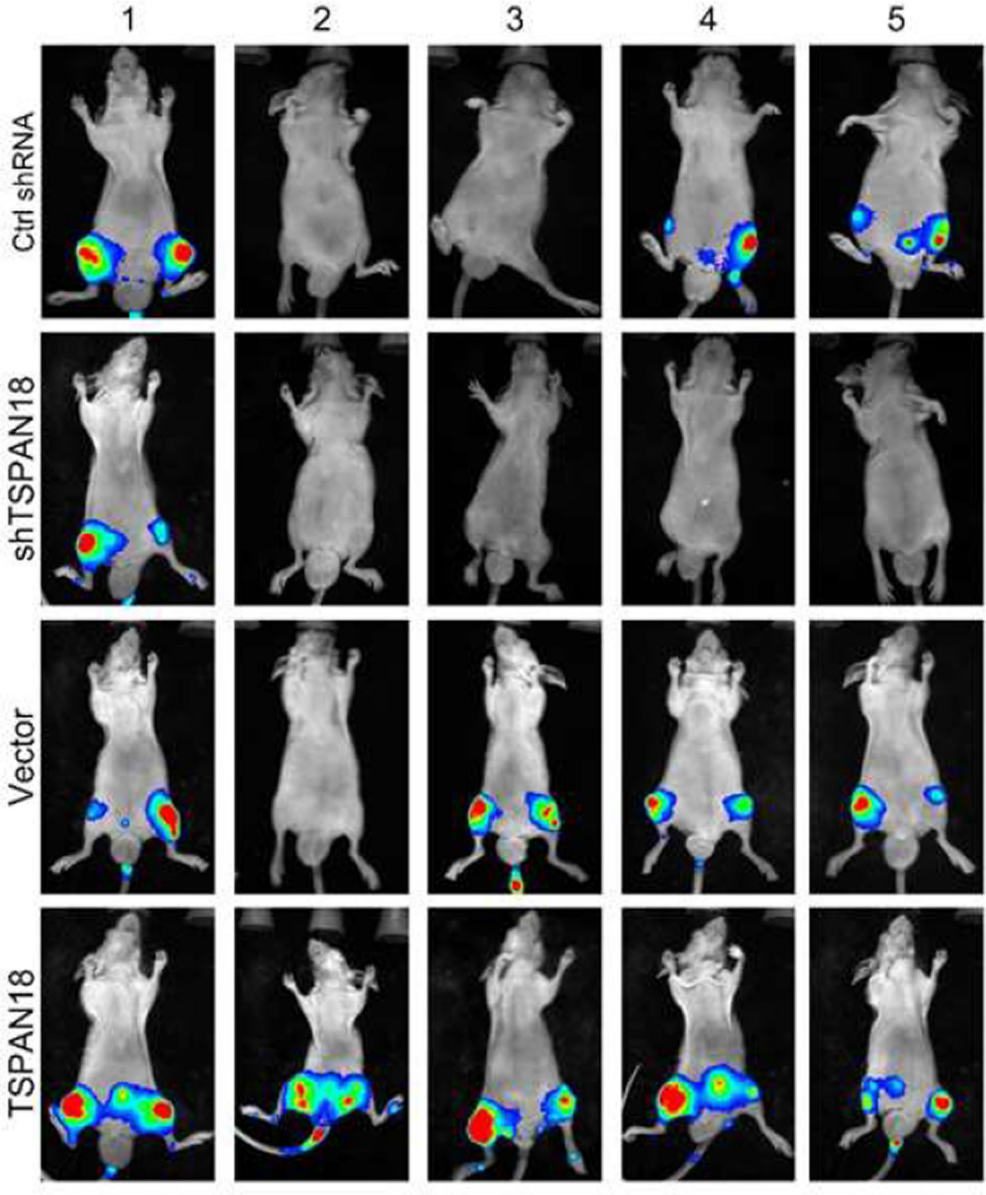
The representative bioluminescence images of the mice after 6 weeks of inoculations with indicated PC-3 cells

**Correct Figure S13**

**Supplemental Fig. 13:**
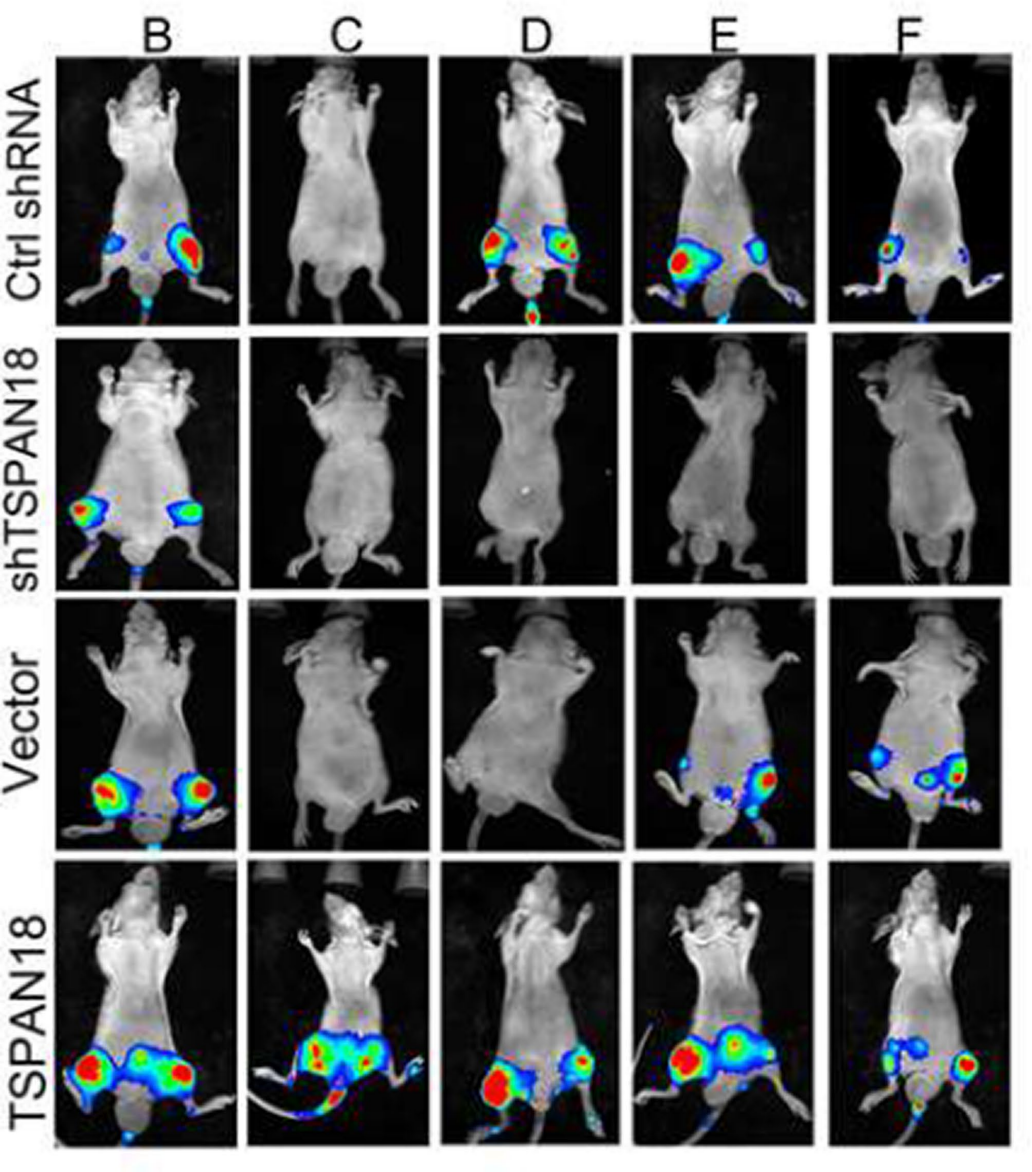
The representative bioluminescence images of the mice after 6 weeks of inoculations with indicated PC-3 cells

The corrections do not compromise the validity of the conclusions and the overall content of the article. The original article [1] has been updated.

## Electronic supplementary material

Below is the link to the electronic supplementary material.

Supplementary Material 1
