# Supplementary material for: TSPAN18 facilitates bone metastasis of prostate cancer by protecting STIM1 from TRIM32-mediated ubiquitination
Source: J Exp Clin Cancer Res. 2023 Aug 5;42:195. doi: 10.1186/s13046-023-02764-4 (PMC10403854; doi:10.1186/s13046-023-02764-4)
Supplement: Supplementary file 1 — Additional file 1: Supplemental Table 1. The antibodies used in this study are listed as follows. Supplemental Table 2. The primers used in real time qPCR are listed as follows. Supplemental Table 3. Basic characteristics of prostate cancer patients in two cohorts. Supplemental Table 4. Univariate analysis of prognostic factors correlated with OS and CSS. Supplemental Table 5. Multivariate analysis of prognostic factors correlated with OS and CSS. Supplemental Figure 1. (a) Co-IPanalysis of interaction between exogenous STIM1 and exogenous TSPAN18 in HEK-293Tcells, transfected with Flag-TSPAN18 and Myc-STIM1 plasmid using anti-Mycantibody(left) or anti-Flag antibody (right). (b) Co-IP analysis of interaction between Flag-TSPAN18 and Myc-Orai1 using anti-Flag antibody (left) or anti-Mycantibody (right) in HEK-293T cells. (c)Co-IP analysis of interaction between Flag-TSPAN18 and Myc-Orai1 using anti-Flag antibody in Flag-TSPAN18 overexpressing DU145 cells. Supplemental Figure 2. The Western Blot analysis of TSPAN18 and STIM1 protein level in DU145 and PC-3 cells treated as indicated. The values are expressed as the mean ± s.d. of three independent experiments. **p<0.01, ***p < 0.001, ANOVA with post hoctest or Student’s t test. Supplemental Figure 3. The qRT-PCR analysis of TSPAN18 and STIM1 mRNA level in DU145 and PC-3 cells treatedas indicated. The values are expressed as the mean ± s.d.of three independent experiments. **p<0.01, ***p < 0.001,ANOVA with post hoc test or Student’s t test. Supplemental Figure 4. The Western Blot analysis of TSPAN18 and STIM1 protein level in DU145 and PC-3 cells treated asindicated. The values are expressed as the mean ± s.d.of three independent experiments. **p<0.01, ***p < 0.001,ANOVA with post hoc test. Baf: Bafilomycin, ns: no significance. Supplemental Figure 5. The representative peptide of TRIM32(a) or MIB1 (b) from mass spectrometry. Supplemental Figure 6. (a)Co-IP analysis of interaction between exogenous STIM1 and exogenous [file 13046_2023_2764_MOESM1_ESM.docx]

**Tables**

**Supplemental Table 1.** The antibodies used in this study are listed as follows.

| Antibodies | Source | Identifier | Application(dilution) |
| --- | --- | --- | --- |
| Anti-STIM1 | CST | Cat#5668 | WB (1:1000), IF (1:800), IP |
| Anti-STIM1 | Proteintech | Cat#11565-1-AP | IHC (1:200) |
| Anti-TSPAN18 | Thermo Scientific | Cat#PA5-48957 | WB (1:500), IHC (1:100) |
| Anti-Flag | CST | Cat#14793 | WB (1:1000), IF (1:400), IP |
| Anti-Myc | CST | Cat#2276 | WB (1:1000), IP |
| Anti-His | CST | Cat#12698 | WB (1:1000), IP |
| Anti-HA | CST | Cat#3724 | WB (1:1000) |
| Anti-E-cadherin | CST | Cat#14472 | WB (1:1000), IF (1:200) |
| Anti-N-cadherin | CST | Cat#13116 | WB (1:1000), IF (1:200) |
| Anti-Vinculin | Abcam | Cat#ab129002 | IF (1:100) |
| Anti-TRIM32 | GeneTex | Cat#113937 | WB (1:1000) |
| Anti-MIB1 | Proteintech | Cat#11893-1-AP | WB (1:1000) |
| Anti-GAPDH | CST | Cat#5174 | WB (1:5000) |
| Normal rabbit IgG | CST | Cat#2729 | IP |
| Rabbit IgG (H+L) | CST | Cat#14708 | IP |
| Mouse IgG (H+L) | CST | Cat#14709 | IP |

Abbreviation: CST, Cell Signaling Technology; WB, Western blot; IF, Immunofluorescence; IP, Immunoprecipitation; IHC, Immunohistochemistry.

**Supplemental Table 2.** The primers used in real time qPCR are listed as follows.

| Primer Name | Sequence 5’-3’ |
| --- | --- |
| TSPAN18 Forward | TTCTTCACCAAGGAGCTCACC |
| TSPAN18 Reverse | CTCCGGCACCTCTTCACTATC |
| STIM1 Forward | CTTGTCCATGCAGTCCCCTAG |
| STIM1 Reverse | GTGGTGATGGAAGAGGAGCAA |
| GPADH Forward | CAAGGCTGAGAACGGGAAG |
| GPADH Reverse | TGAAGACGCCAGTGGACTC |

**Supplemental Table 3.** Basic characteristics of prostate cancer patients in two cohorts

| Variables |  | Training cohort (n=126) |  | Validation cohort (n=113) |
| --- | --- | --- | --- | --- |
|  |  | Number of cases (%) |  | Number of cases (%) |
| Age (y, Mean range) | 66(42-89)  <66  ≥66 | 59(46.8%)  67(53.2%) |  | 41(36.3%)  72(63.7%) |
| T stage | T2  T3  T4 | 76(60.3%)  30(23.8%)  20(15.9%) |  | 80(70.8%)  28(24.8%)  5(4.4%) |
| N stage | N0  N1 | 116(92.1%)  10(7.9%) |  | 110(97.3%)  3(2.7%) |
| M stage | M0  M1 | 116(92.1%)  10(7.9%) |  | 105(92.9%)  8(7.1%) |
| Gleason score | 10  9  8  7(4+3)  7(3+4)  ≤6 | 4(3.2%)  23(18.2%)  16(12.7%)  18(14.3%)  24(19.0%)  41(32.6%) |  | 3(2.7%)  11(9.7%)  9(8.0%)  36(31.9%)  28(24.8%)  26(23.0%) |
| TSPAN18 | Low  High | 78(61.9%)  48(38.1%) |  | 70(61.9%)  43(38.1%) |

**Supplemental Table 4.** Univariate analysis of prognostic factors correlated with OS and CSS

| Variables | OS | | | | |  | CSS | | | | |
| --- | --- | --- | --- | --- | --- | --- | --- | --- | --- | --- | --- |
|  | Cohort 1 | |  | Cohort 2 | |  | Cohort 1 | |  | Cohort 2 | |
|  | HR  (95%CI) | *p*-value |  | HR  (95%CI) | *p*-value |  | HR  (95%CI) | *p*-value |  | HR  (95%CI) | *p*-  value |
| Age (y)  ≥66/<66 | 1.94  0.98-3.88 | 0.059 |  | 1.62  0.63-4.14 | 0.341 |  | 1.69  0.76-3.76 | 0.197 |  | 1.21  0.43-3.41 | 0.713 |
| Gleason score 7(4+3)-10/6-7(3+4) | 2.58  1.27-5.24 | **0.009** |  | 7.28  2.14-24.67 | **0.001** |  | 2.60  1.13-5.98 | **0.025** |  | 7.06  1.59-31.39 | **0.010** |
| Tumor stage  T3-4/T2 | 2.14  1.10-4.17 | **0.025** |  | 5.50  2.30-13.16 | **<0.001** |  | 2.46  1.11-5.43 | **0.026** |  | 6.03  2.06-17.71 | **0.001** |
| Distal metastasis  Present/Absent | 5.83  2.63-12.93 | **<0.001** |  | 4.43  1.63-12.03 | **0.003** |  | 6.17  2.45-15.52 | **<0.001** |  | 7.27  2.48-21.30 | **<0.001** |
| TSPAN18  High/Low | 2.35  1.21-4.54 | **0.011** |  | 2.77  1.18-6.59 | **0.019** |  | 2.39  1.08-5.30 | **0.031** |  | 3.75  1.28-11.00 | **0.016** |

Abbreviations: OS, overall survival; CSS, cancer-specific survival; HR hazard ratio, CI confidence interval; *p*-value<0.05 marked in bold font shows statistically significant.

**Supplemental Table 5.** Multivariate analysis of prognostic factors correlated with OS and CSS

| Variables | OS | | | | |  | CSS | | | | |
| --- | --- | --- | --- | --- | --- | --- | --- | --- | --- | --- | --- |
|  | Cohort 1 | |  | Cohort 2 | |  | Cohort 1 | |  | Cohort 2 | |
|  | HR  (95%CI) | *p*-value |  | HR  (95%CI) | *p*-value |  | HR  (95%CI) | *p*-value |  | HR  (95%CI) | *p*-  value |
| Gleason score 7(4+3)-10/6-7(3+4) | 1.66  0.77-3.57 | 0.192 |  | 4.76  1.35-16.77 | **0.015** |  | 1.56  0.63-3.85 | 0.333 |  | 3.88  0.82-18.41 | 0.088 |
| Tumor stage  T3-4/T2 | 1.84  0.92-3.67 | 0.085 |  | 4.05  1.61-10.18 | **0.003** |  | 1.96  0.85-4.49 | 0.114 |  | 3.80  1.19-12.14 | 0.025 |
| Distal metastasis  Present/Absent | 5.48  2.41-12.43 | **<0.001** |  | 1.24  0.42-3.67 | 0.699 |  | 6.28  2.40-16.40 | **<0.001** |  | 2.07  0.62-6.86 | 0.236 |
| TSPAN18  High/Low | 2.27  1.11-4.67 | **0.025** |  | 1.98  0.82-4.79 | 0.130 |  | 2.43  1.03-5.77 | **0.044** |  | 2.43  0.80-7.43 | 0.120 |

Abbreviations: OS, overall survival; CSS, cancer-specific survival; HR hazard ratio, CI confidence interval; *p*-value<0.05 marked in bold font shows statistically significant.

**
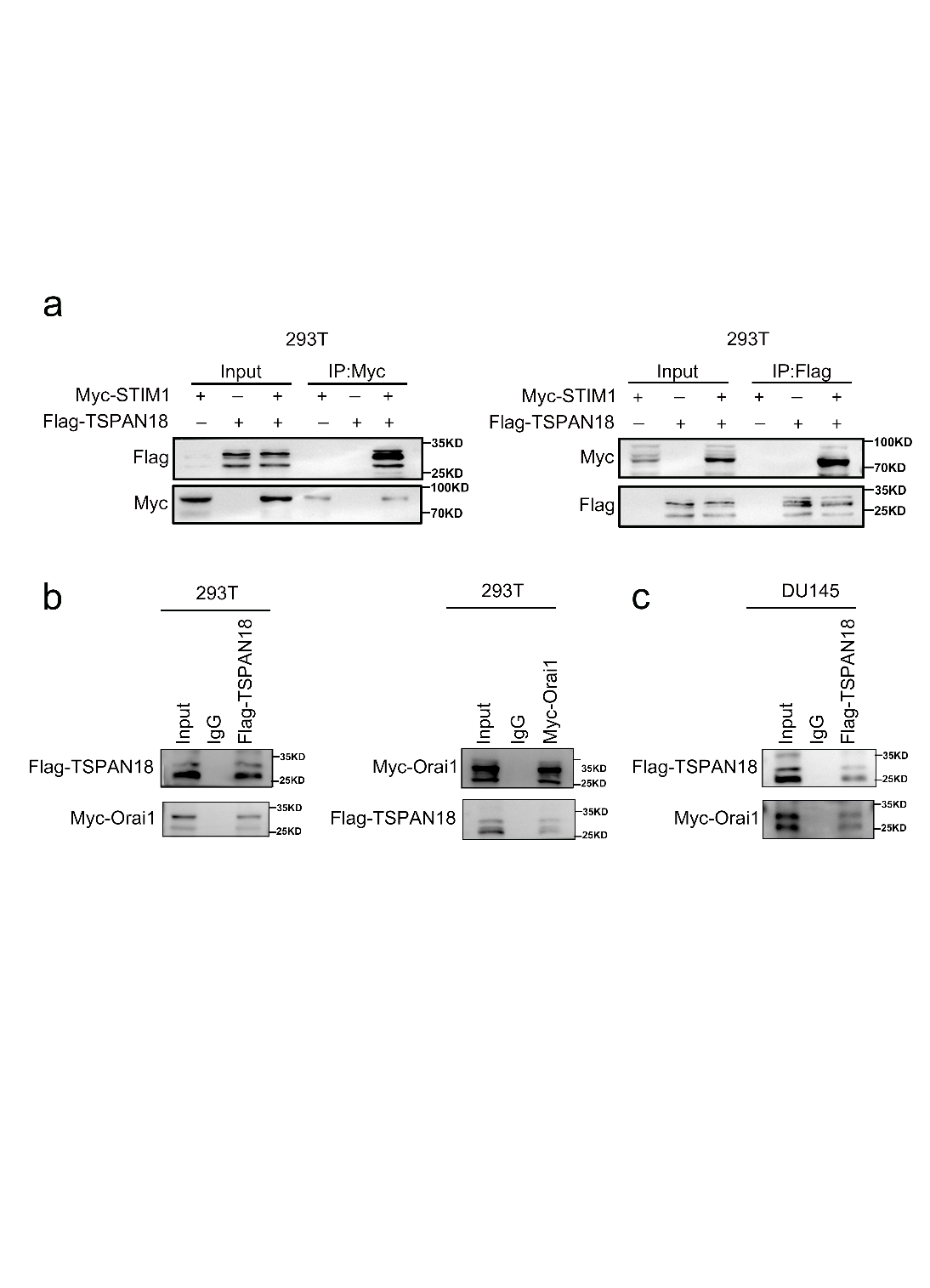
Figures and Legends**


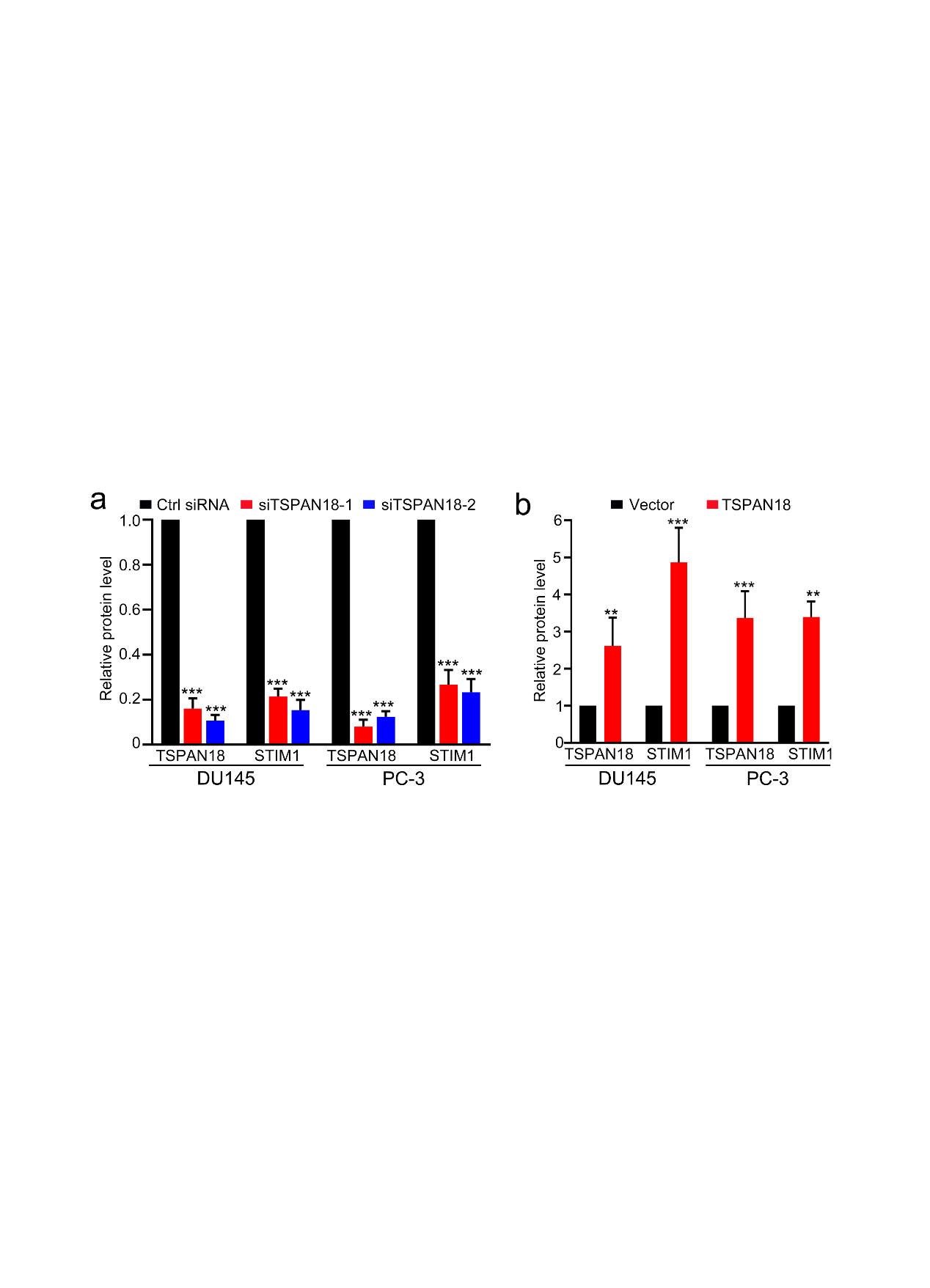
**Supplemental Figure 1** (a) Co-IP analysis of interaction between exogenous STIM1 and exogenous TSPAN18 in HEK-293T cells, transfected with Flag-TSPAN18 and Myc-STIM1 plasmid using anti-Myc antibody(left) or anti-Flag antibody (right). (b) Co-IP analysis of interaction between Flag-TSPAN18 and Myc-Orai1 using anti-Flag antibody (left) or anti-Myc antibody (right) in HEK-293T cells. (c) Co-IP analysis of interaction between Flag-TSPAN18 and Myc-Orai1 using anti-Flag antibody in Flag-TSPAN18 overexpressing DU145 cells.

**Supplemental Figure 2** The Western Blot analysis of TSPAN18 and STIM1 protein level in DU145 and PC-3 cells treated as indicated. The values are expressed as the mean ± s.d. of three independent experiments. ***p*<0.01, ****p* < 0.001, ANOVA with post hoc test or Student’s t test.


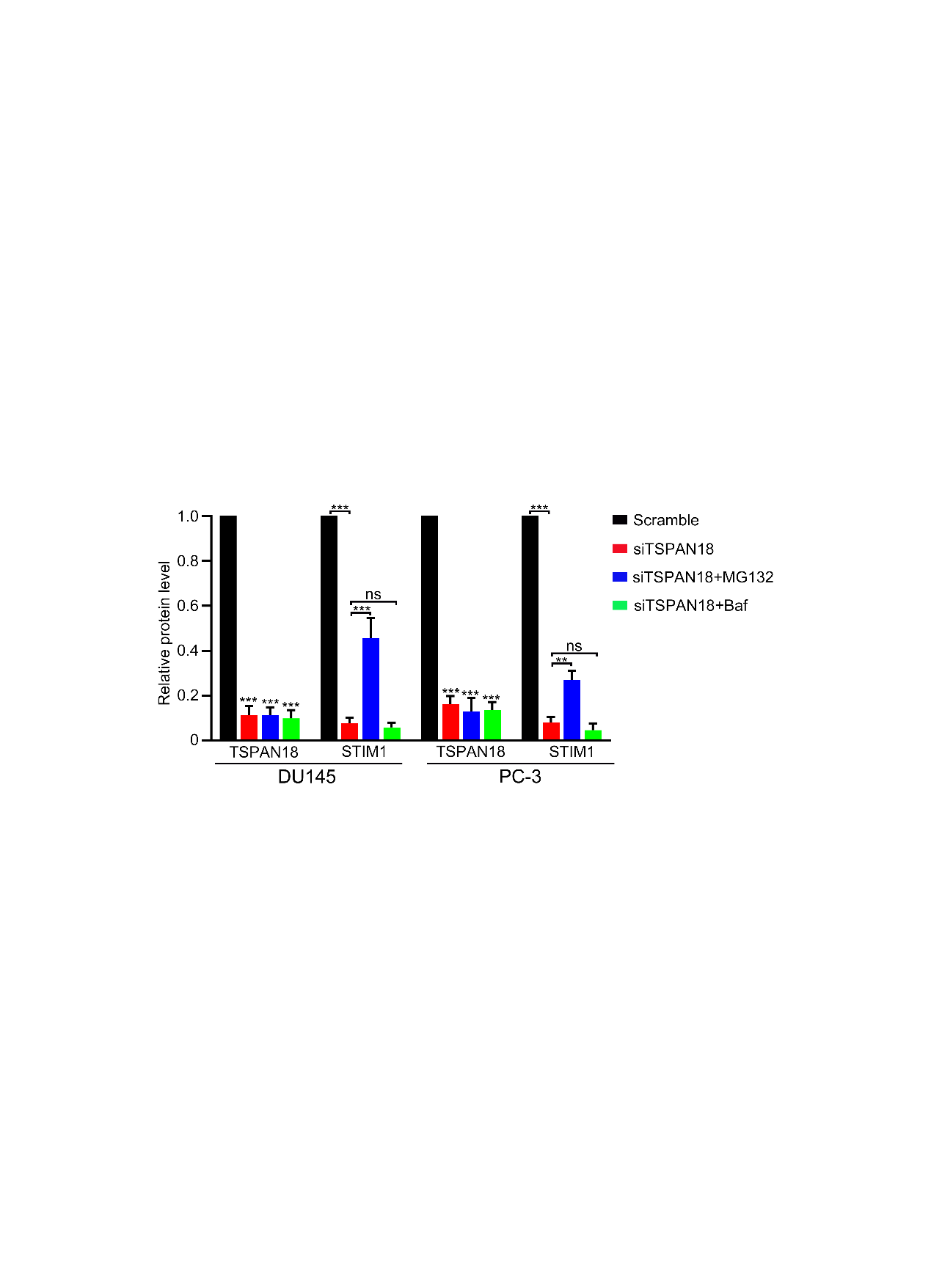

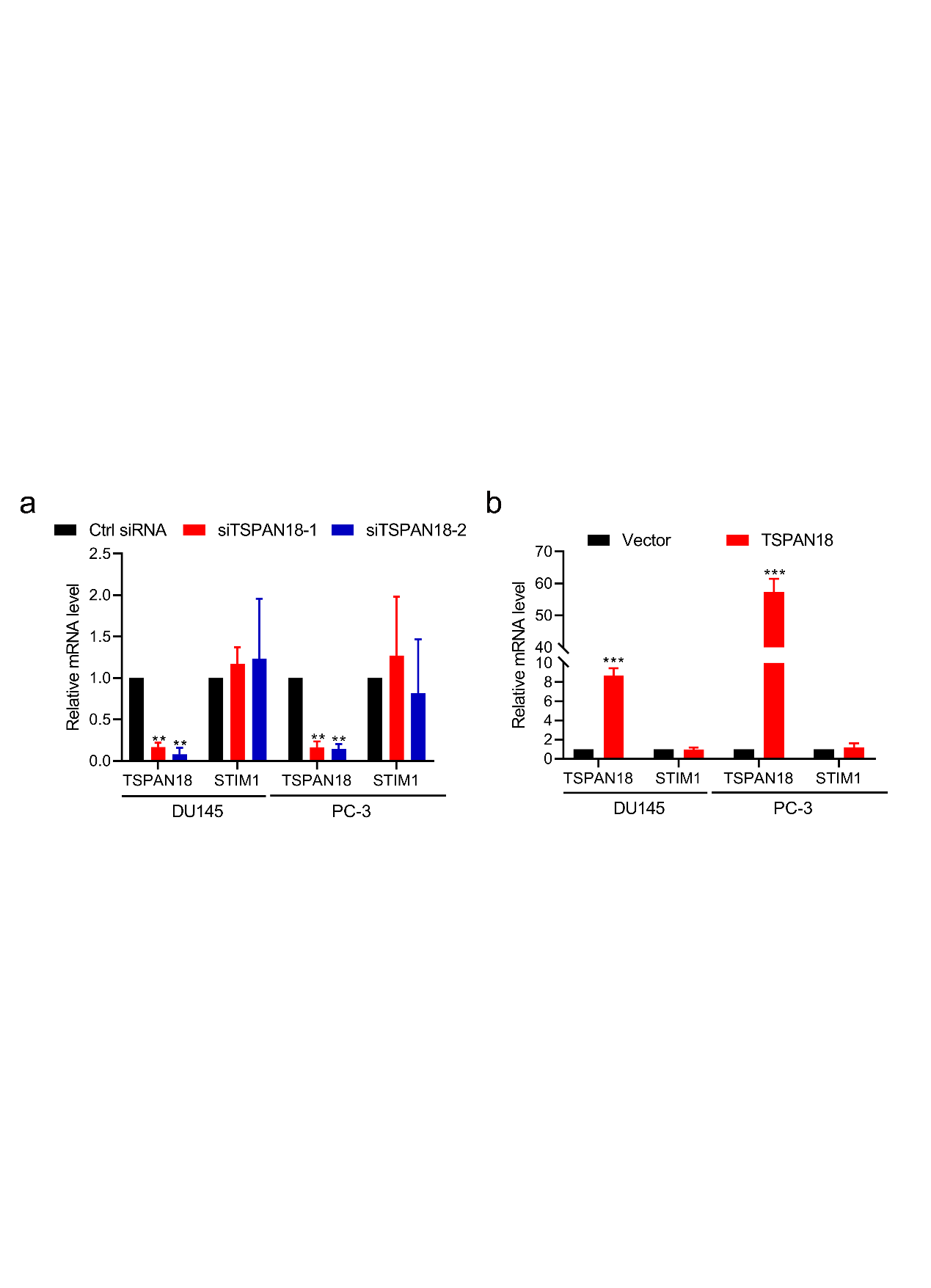
**Supplemental Figure 3** The qRT-PCR analysis of TSPAN18 and STIM1 mRNA level in DU145 and PC-3 cells treated as indicated. The values are expressed as the mean ± s.d. of three independent experiments. ***p*<0.01, ****p* < 0.001, ANOVA with post hoc test or Student’s *t* test.

**Supplemental Figure 4** The Western Blot analysis of TSPAN18 and STIM1 protein level in DU145 and PC-3 cells treated as indicated. The values are expressed as the mean ± s.d. of three independent experiments. ***p*<0.01, ****p* < 0.001, ANOVA with post hoc test. Baf: Bafilomycin, ns: no significance.


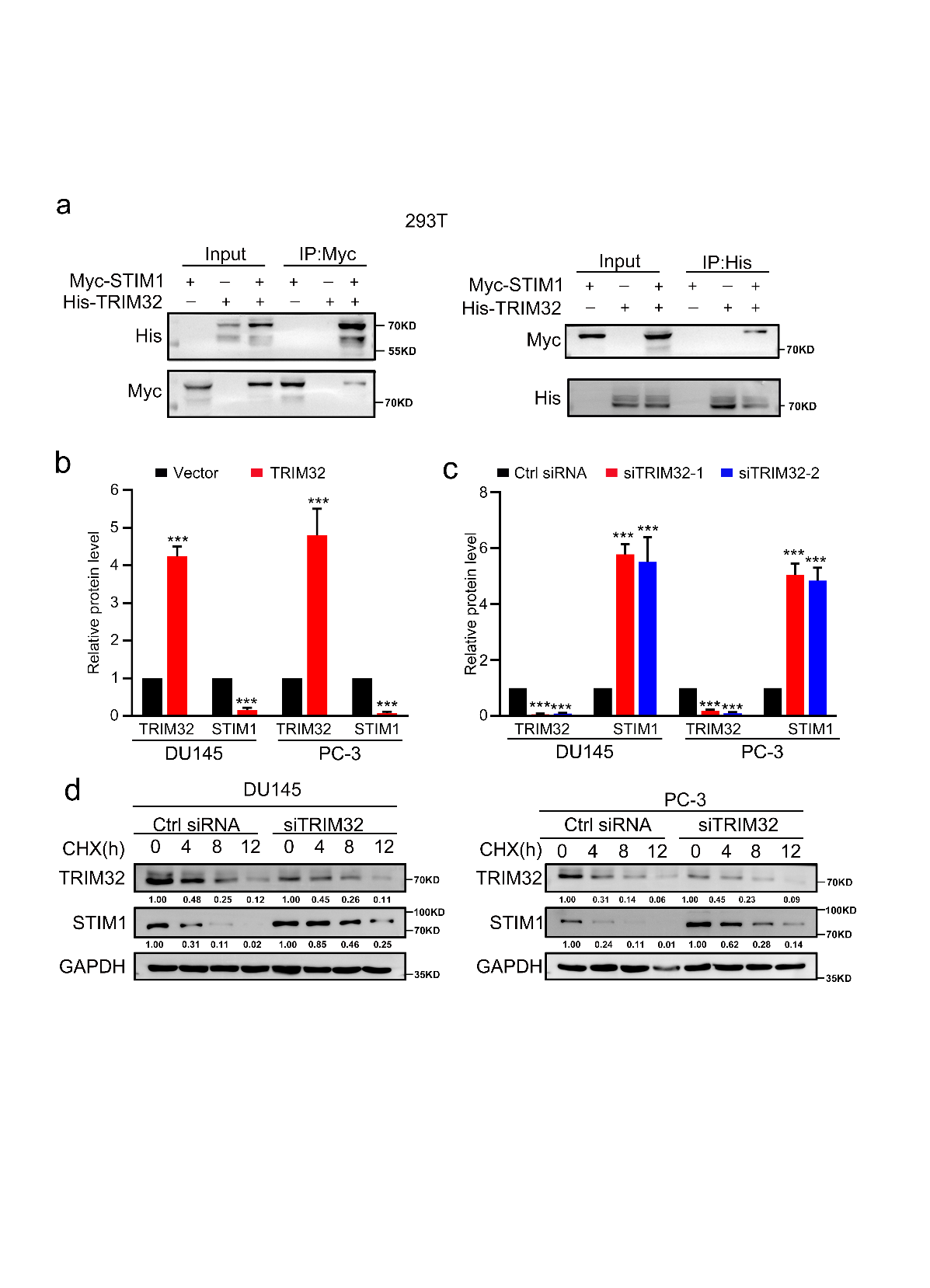

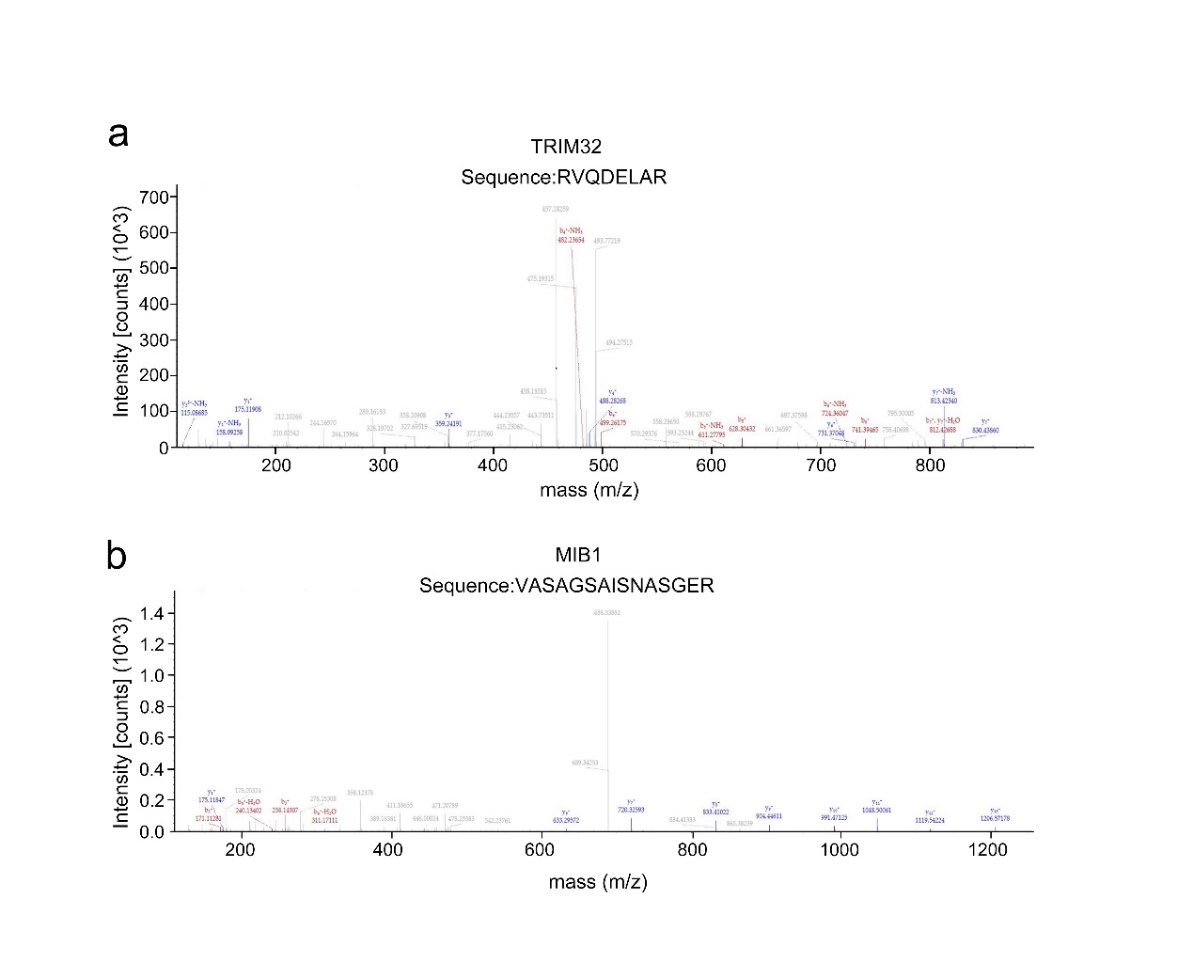
**Supplemental Figure 5** The representative peptide of TRIM32 (a) or MIB1 (b) from mass spectrometry.

**Supplemental Figure 6** (a) Co-IP analysis of interaction between exogenous STIM1 and


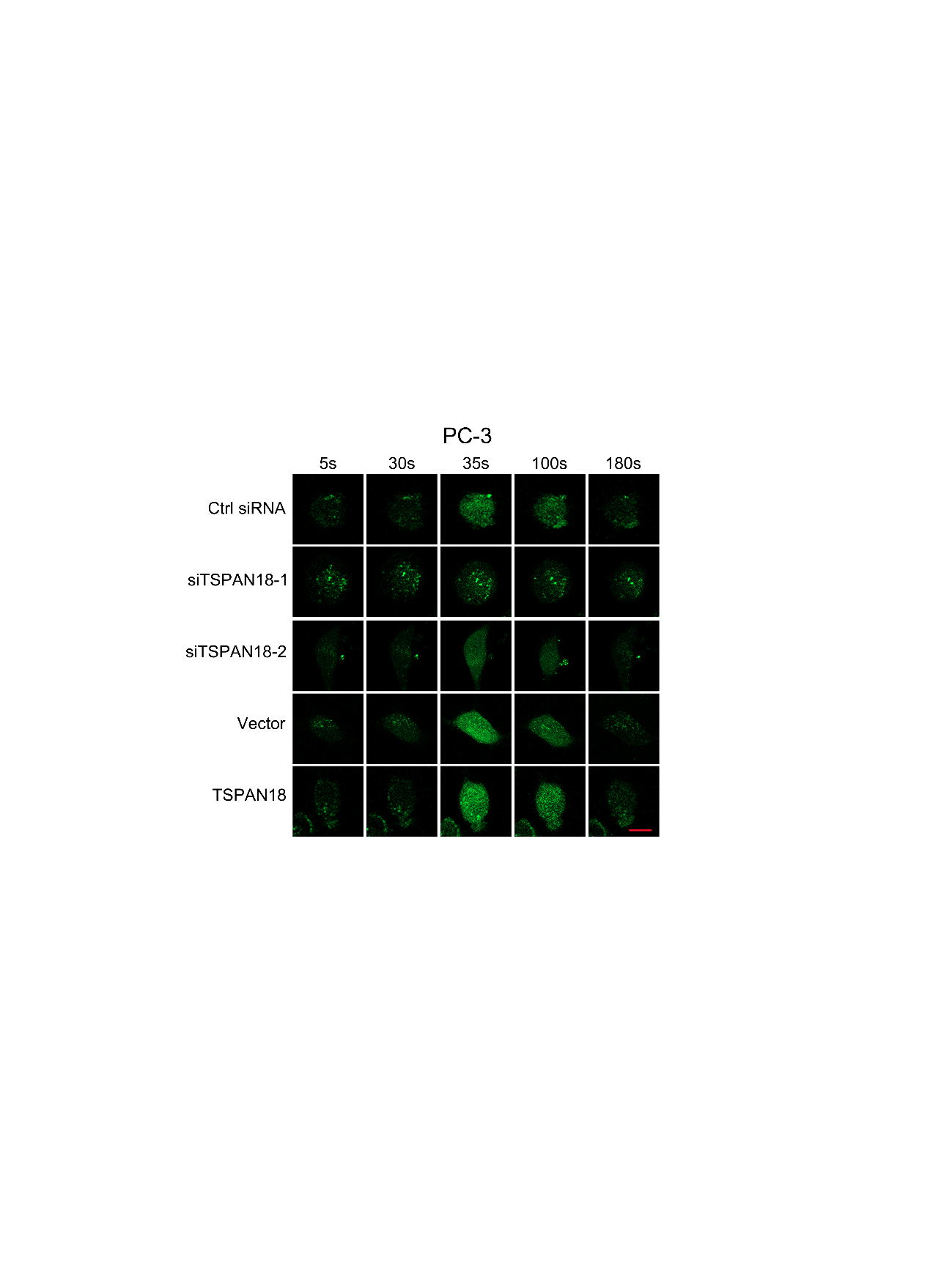
exogenous TRIM32 in HEK-293T cells transfected with His-TRIM32 plasmid and Myc-STIM1 plasmid using anti-Myc antibody(left) or anti-His antibody (right). (b-c) The Western Blot analysis of TRIM32 and STIM1 protein level in DU145 and PC-3 cells treated as indicated. The values are expressed as the mean ± s.d. of three independent experiments. ****p* < 0.001, Student’s t test or ANOVA with post hoc test. (d) The protein level of STIM1 in DU145 and PC-3 cells transfected with scramble or si-TRIM32 were monitored by WB at indicated times after cycloheximide (CHX, 20μg/mL).


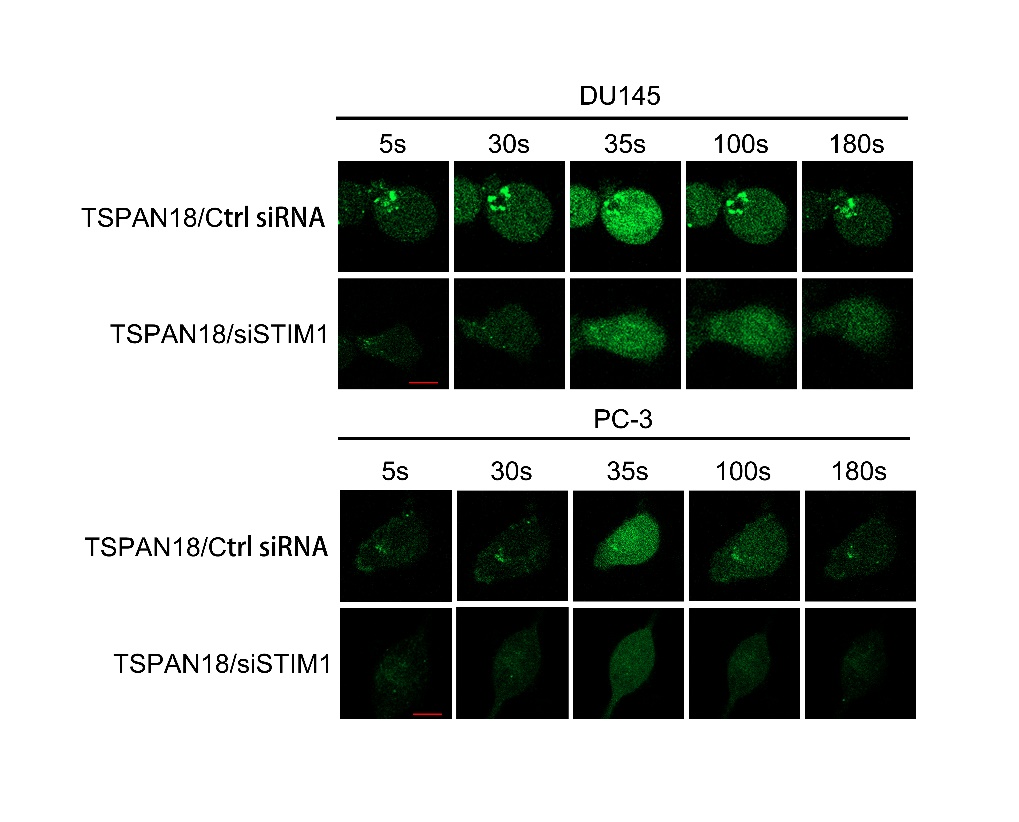
**Supplemental Figure 7** The representative time-lapse images of cytosolic Ca^2+^ level within PC-3 cells transfected with indicated siRNAs or plasmids. Scale bars: red, 50 μm.

**Supplemental Figure 8** The representative time-lapse images of cytosolic Ca^2+^ level within

indicated cells. Scale bars: red, 50 μm.


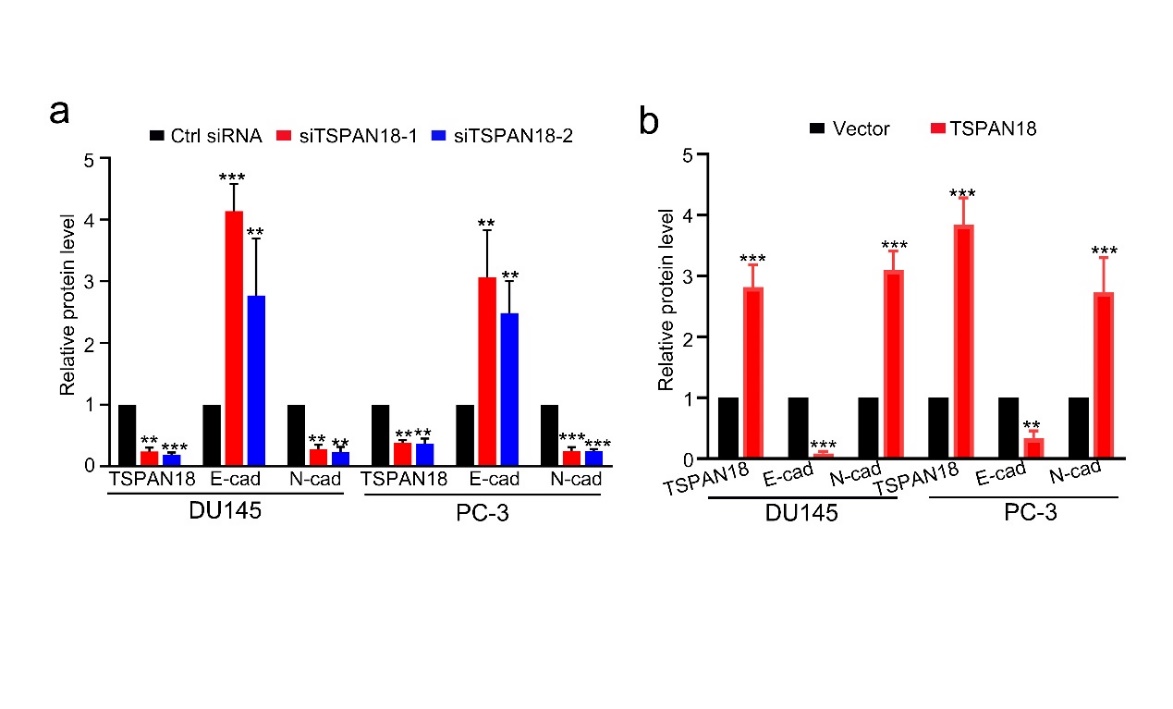

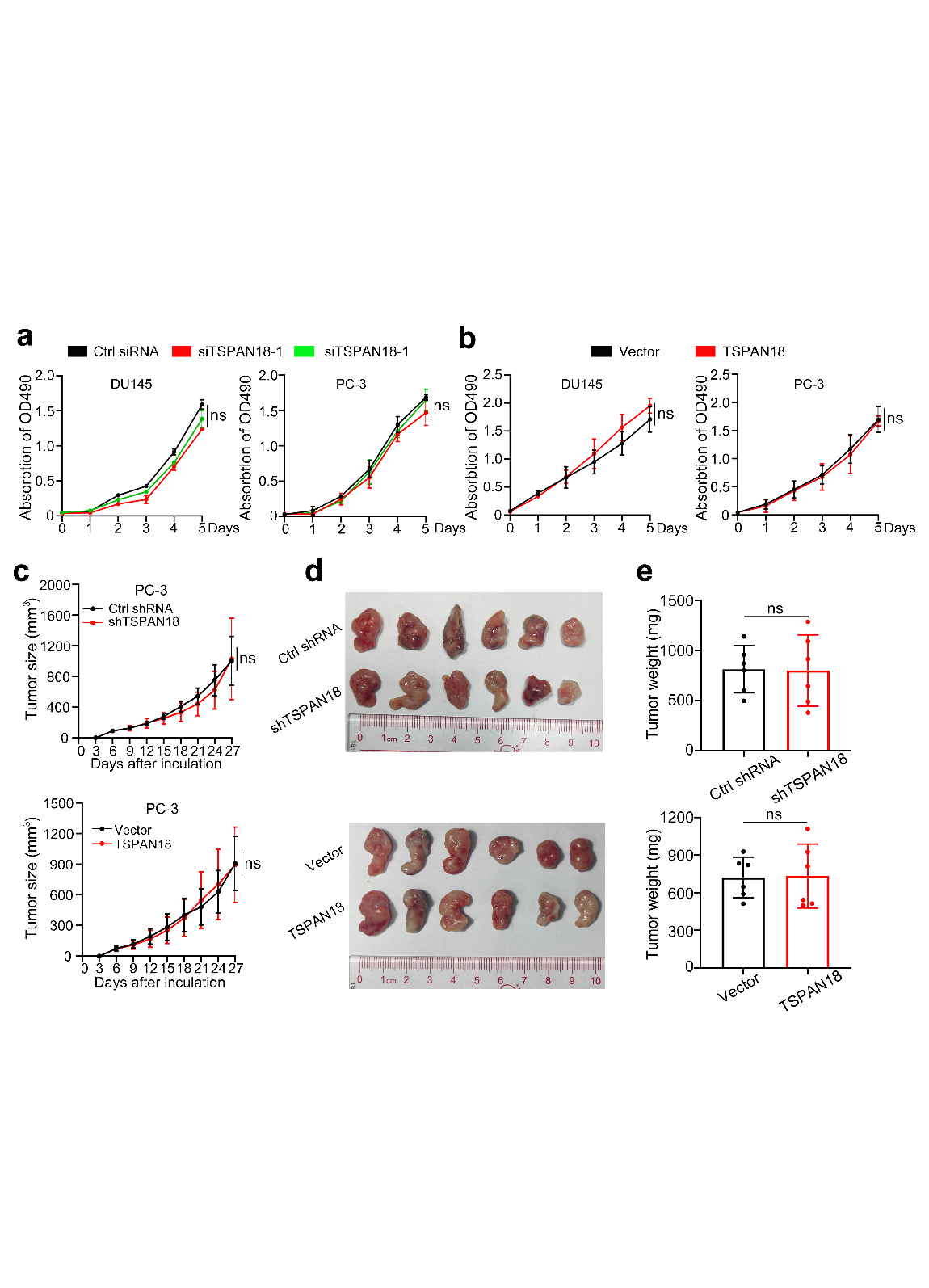
**Supplemental Figure 9** (a-b) Cell viability was evaluated in TSPAN18 knockdown or overexpressing DU145 and PC-3 cells. (c) Tumor growth curves are summarized in the line chart. The average tumor volume is expressed as the mean ± SD of six mice. (d) Representative images of the tumors of TSPAN18 knockdown or overexpression groups and their respective controls. (e) Tumor weights were measured after the tumors were surgically dissected. ns: no significance.

**Supplemental Figure 10** The Western Blot analysis of E-cadherin and N-cadherin protein level in DU145 and PC-3 cells treated as indicated. The values are expressed as the mean ± s.d. of three independent experiments. ***p*<0.01, ****p* < 0.001, ANOVA with post hoc test or

Student’s *t* test.


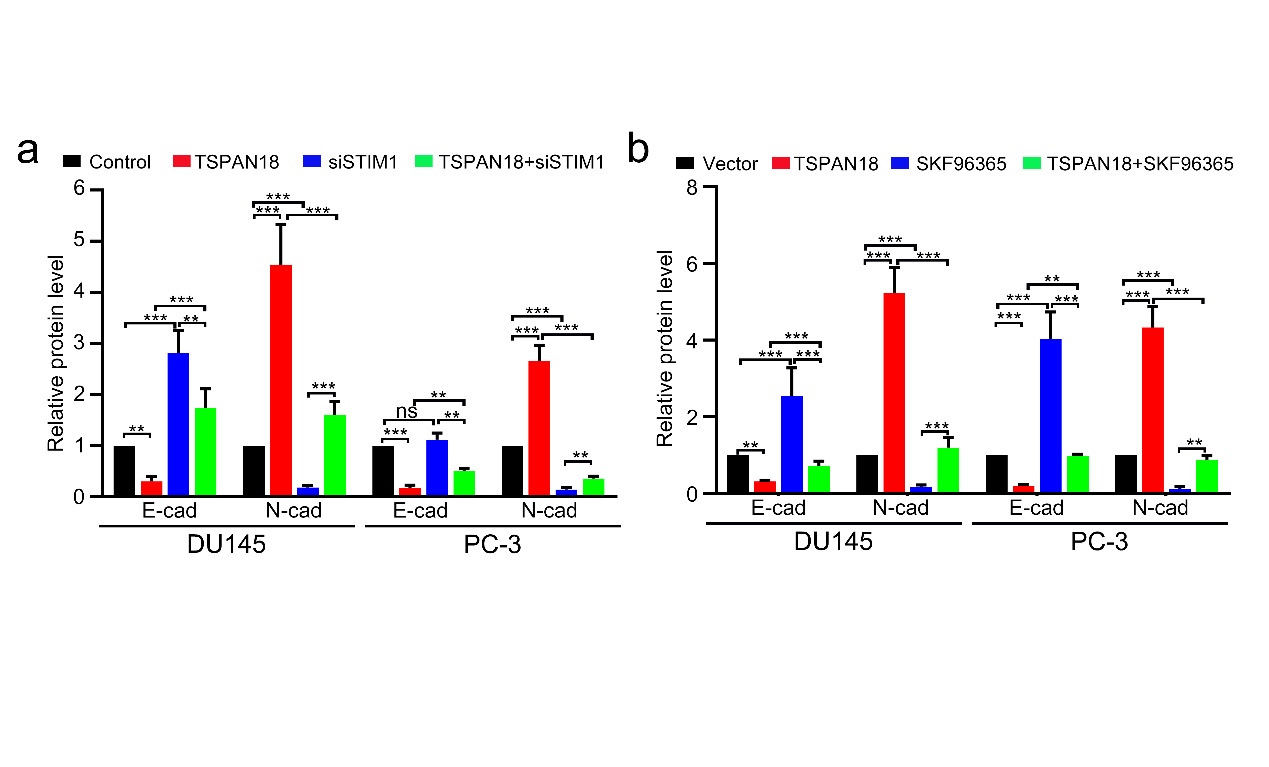

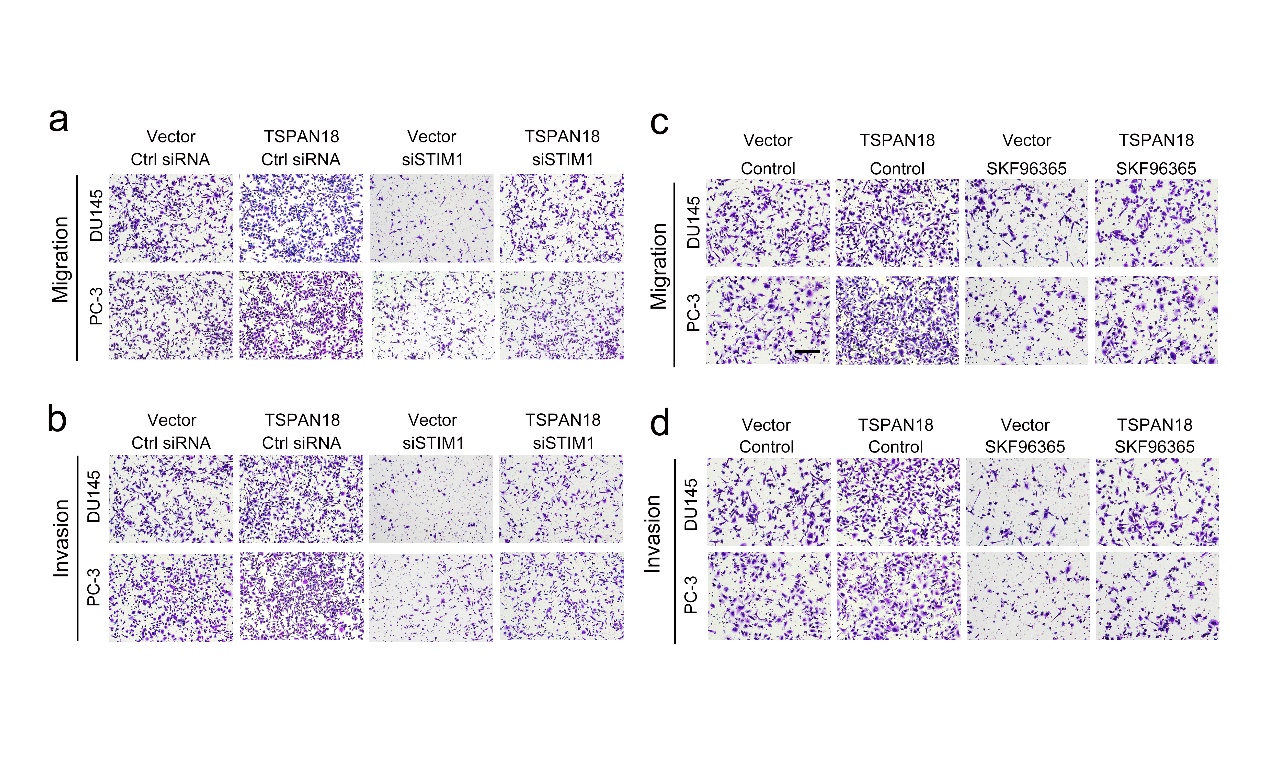
**Supplemental Figure 11** The representative images of migration and invasion assays using TSPAN18-overexpressing or control DU145 and PC-3 cells transfected with indicated siRNAs (a, b) or treated with SKF96365 or DMSO (c, d).

**Supplemental Figure 12** The Western Blot analysis of N-cadherin and E-cadherin protein level in DU145 and PC-3 cells treated as indicated. The values are expressed as the mean ± s.d. of three independent experiments. ***p*<0.01, ****p* < 0.001, ANOVA with post hoc test. ns: no significance.


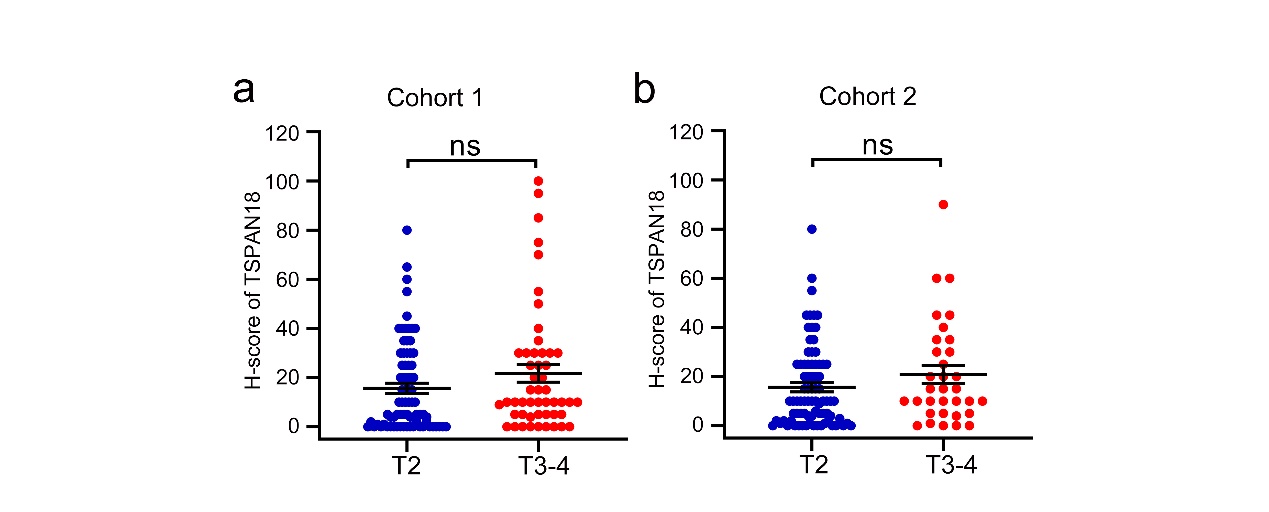

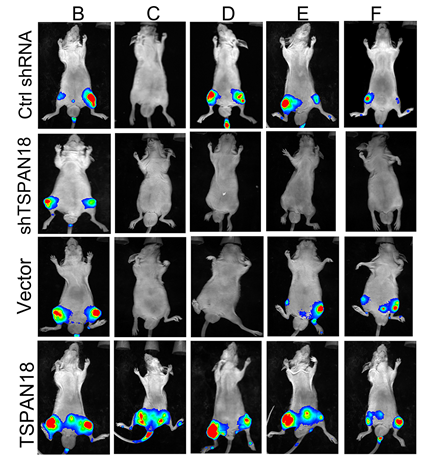


**Supplemental Figure 13** The representative bioluminescence images of the mice after 6 weeks of inoculations with indicated PC-3 cells.

**Supplemental Figure 14** The expression difference of TSPAN18 between low T stage PCa tissues and high T stage PCa tissues in Cohort 1 (a) and Cohort 2 (b). ns: no significance. Student’s *t* test.
