# Supplementary material for: Correction: TSPAN18 facilitates bone metastasis of prostate cancer by protecting STIM1 from TRIM32-mediated ubiquitination
Source: J Exp Clin Cancer Res. 2025 Apr 10;44:116. doi: 10.1186/s13046-025-03373-z (PMC11984017; doi:10.1186/s13046-025-03373-z)
Supplement: Supplementary file 1 — Supplementary Material 1 [file 13046_2025_3373_MOESM1_ESM.docx]

**Tables**

**Supplemental Table 1.** The antibodies used in this study are listed as follows.

| Antibodies | Source | Identifier | Application(dilution) |
| --- | --- | --- | --- |
| Anti-STIM1 | CST | Cat#5668 | WB (1:1000), IF (1:800), IP |
| Anti-STIM1 | Proteintech | Cat#11565-1-AP | IHC (1:200) |
| Anti-TSPAN18 | Thermo Scientific | Cat#PA5-48957 | WB (1:500), IHC (1:100) |
| Anti-Flag | CST | Cat#14793 | WB (1:1000), IF (1:400), IP |
| Anti-Myc | CST | Cat#2276 | WB (1:1000), IP |
| Anti-His | CST | Cat#12698 | WB (1:1000), IP |
| Anti-HA | CST | Cat#3724 | WB (1:1000) |
| Anti-E-cadherin | CST | Cat#14472 | WB (1:1000), IF (1:200) |
| Anti-N-cadherin | CST | Cat#13116 | WB (1:1000), IF (1:200) |
| Anti-Vinculin | Abcam | Cat#ab129002 | IF (1:100) |
| Anti-TRIM32 | GeneTex | Cat#113937 | WB (1:1000) |
| Anti-MIB1 | Proteintech | Cat#11893-1-AP | WB (1:1000) |
| Anti-GAPDH | CST | Cat#5174 | WB (1:5000) |
| Normal rabbit IgG | CST | Cat#2729 | IP |
| Rabbit IgG (H+L) | CST | Cat#14708 | IP |
| Mouse IgG (H+L) | CST | Cat#14709 | IP |

Abbreviation: CST, Cell Signaling Technology; WB, Western blot; IF, Immunofluorescence; IP, Immunoprecipitation; IHC, Immunohistochemistry.

**Supplemental Table 2.** The primers used in real time qPCR are listed as follows.

| Primer Name | Sequence 5’-3’ |
| --- | --- |
| TSPAN18 Forward | TTCTTCACCAAGGAGCTCACC |
| TSPAN18 Reverse | CTCCGGCACCTCTTCACTATC |
| STIM1 Forward | CTTGTCCATGCAGTCCCCTAG |
| STIM1 Reverse | GTGGTGATGGAAGAGGAGCAA |
| GPADH Forward | CAAGGCTGAGAACGGGAAG |
| GPADH Reverse | TGAAGACGCCAGTGGACTC |

**Supplemental Table 3.** Basic characteristics of prostate cancer patients in two cohorts

| Variables |  | Training cohort (n=126) |  | Validation cohort (n=113) |
| --- | --- | --- | --- | --- |
|  |  | Number of cases (%) |  | Number of cases (%) |
| Age (y, Mean range) | 66(42-89)  <66  ≥66 | 59(46.8%)  67(53.2%) |  | 41(36.3%)  72(63.7%) |
| T stage | T2  T3  T4 | 76(60.3%)  30(23.8%)  20(15.9%) |  | 80(70.8%)  28(24.8%)  5(4.4%) |
| N stage | N0  N1 | 116(92.1%)  10(7.9%) |  | 110(97.3%)  3(2.7%) |
| M stage | M0  M1 | 116(92.1%)  10(7.9%) |  | 105(92.9%)  8(7.1%) |
| Gleason score | 10  9  8  7(4+3)  7(3+4)  ≤6 | 4(3.2%)  23(18.2%)  16(12.7%)  18(14.3%)  24(19.0%)  41(32.6%) |  | 3(2.7%)  11(9.7%)  9(8.0%)  36(31.9%)  28(24.8%)  26(23.0%) |
| TSPAN18 | Low  High | 78(61.9%)  48(38.1%) |  | 70(61.9%)  43(38.1%) |

**Supplemental Table 4.** Univariate analysis of prognostic factors correlated with OS and CSS

| Variables | OS | | | | |  | CSS | | | | |
| --- | --- | --- | --- | --- | --- | --- | --- | --- | --- | --- | --- |
|  | Cohort 1 | |  | Cohort 2 | |  | Cohort 1 | |  | Cohort 2 | |
|  | HR  (95%CI) | *p*-value |  | HR  (95%CI) | *p*-value |  | HR  (95%CI) | *p*-value |  | HR  (95%CI) | *p*-  value |
| Age (y)  ≥66/<66 | 1.94  0.98-3.88 | 0.059 |  | 1.62  0.63-4.14 | 0.341 |  | 1.69  0.76-3.76 | 0.197 |  | 1.21  0.43-3.41 | 0.713 |
| Gleason score 7(4+3)-10/6-7(3+4) | 2.58  1.27-5.24 | **0.009** |  | 7.28  2.14-24.67 | **0.001** |  | 2.60  1.13-5.98 | **0.025** |  | 7.06  1.59-31.39 | **0.010** |
| Tumor stage  T3-4/T2 | 2.14  1.10-4.17 | **0.025** |  | 5.50  2.30-13.16 | **<0.001** |  | 2.46  1.11-5.43 | **0.026** |  | 6.03  2.06-17.71 | **0.001** |
| Distal metastasis  Present/Absent | 5.83  2.63-12.93 | **<0.001** |  | 4.43  1.63-12.03 | **0.003** |  | 6.17  2.45-15.52 | **<0.001** |  | 7.27  2.48-21.30 | **<0.001** |
| TSPAN18  High/Low | 2.35  1.21-4.54 | **0.011** |  | 2.77  1.18-6.59 | **0.019** |  | 2.39  1.08-5.30 | **0.031** |  | 3.75  1.28-11.00 | **0.016** |

Abbreviations: OS, overall survival; CSS, cancer-specific survival; HR hazard ratio, CI confidence interval; *p*-value<0.05 marked in bold font shows statistically significant.

**Supplemental Table 5.** Multivariate analysis of prognostic factors correlated with OS and CSS

| Variables | OS | | | | |  | CSS | | | | |
| --- | --- | --- | --- | --- | --- | --- | --- | --- | --- | --- | --- |
|  | Cohort 1 | |  | Cohort 2 | |  | Cohort 1 | |  | Cohort 2 | |
|  | HR  (95%CI) | *p*-value |  | HR  (95%CI) | *p*-value |  | HR  (95%CI) | *p*-value |  | HR  (95%CI) | *p*-  value |
| Gleason score 7(4+3)-10/6-7(3+4) | 1.66  0.77-3.57 | 0.192 |  | 4.76  1.35-16.77 | **0.015** |  | 1.56  0.63-3.85 | 0.333 |  | 3.88  0.82-18.41 | 0.088 |
| Tumor stage  T3-4/T2 | 1.84  0.92-3.67 | 0.085 |  | 4.05  1.61-10.18 | **0.003** |  | 1.96  0.85-4.49 | 0.114 |  | 3.80  1.19-12.14 | 0.025 |
| Distal metastasis  Present/Absent | 5.48  2.41-12.43 | **<0.001** |  | 1.24  0.42-3.67 | 0.699 |  | 6.28  2.40-16.40 | **<0.001** |  | 2.07  0.62-6.86 | 0.236 |
| TSPAN18  High/Low | 2.27  1.11-4.67 | **0.025** |  | 1.98  0.82-4.79 | 0.130 |  | 2.43  1.03-5.77 | **0.044** |  | 2.43  0.80-7.43 | 0.120 |

Abbreviations: OS, overall survival; CSS, cancer-specific survival; HR hazard ratio, CI confidence interval; *p*-value<0.05 marked in bold font shows statistically significant.

**
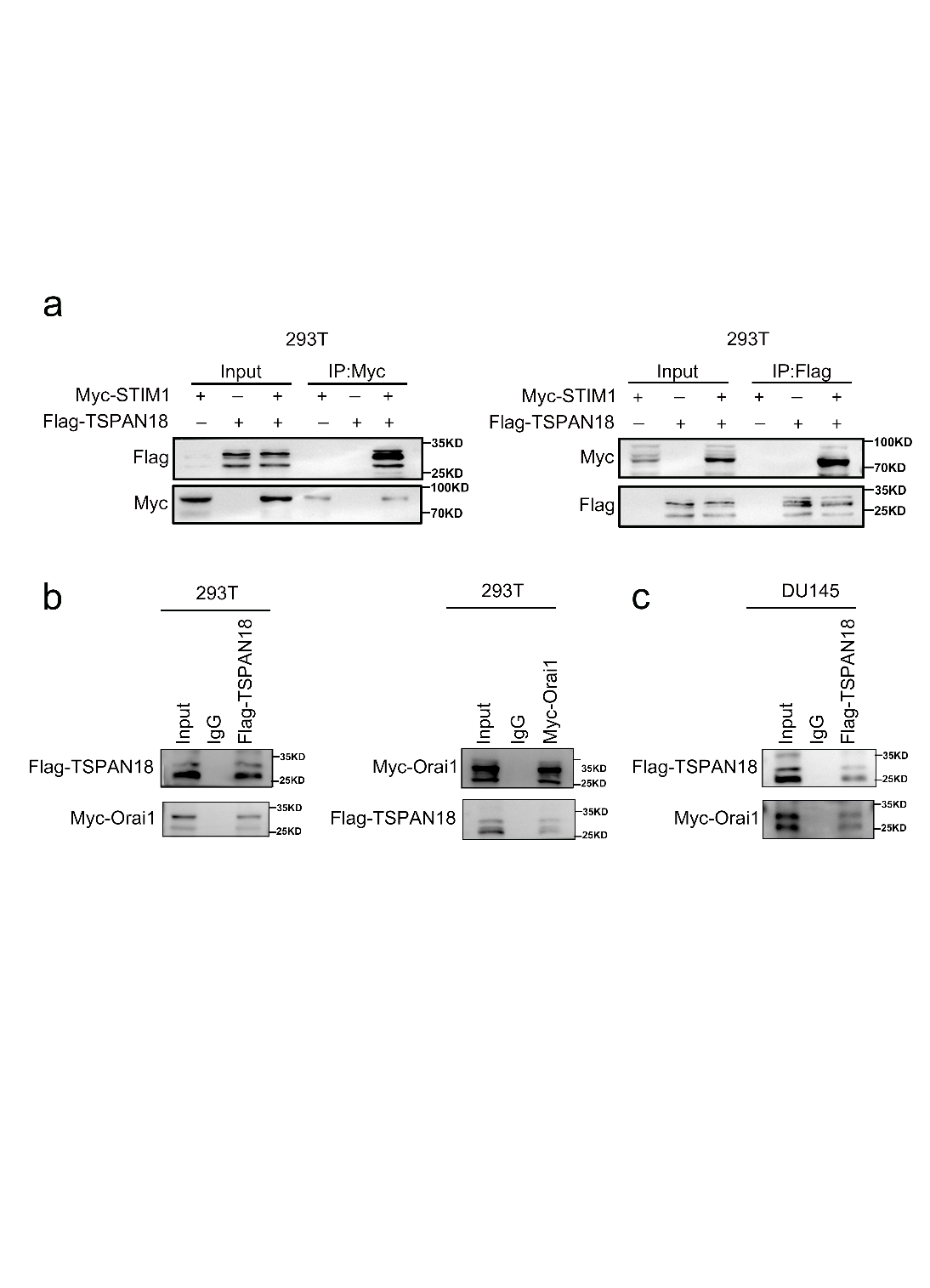
Figures and Legends**


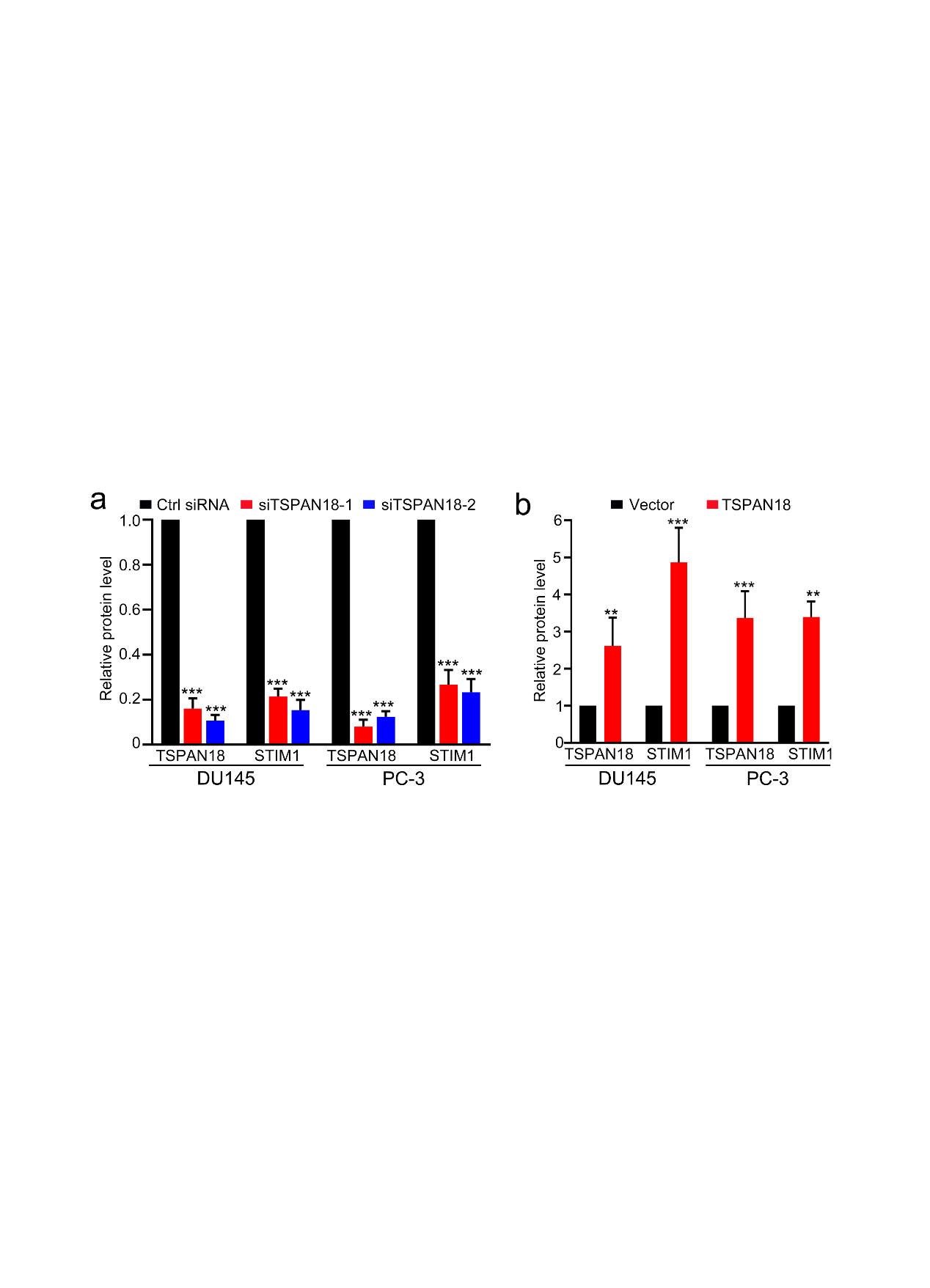
**Supplemental Figure 1** (a) Co-IP analysis of interaction between exogenous STIM1 and exogenous TSPAN18 in HEK-293T cells, transfected with Flag-TSPAN18 and Myc-STIM1 plasmid using anti-Myc antibody(left) or anti-Flag antibody (right). (b) Co-IP analysis of interaction between Flag-TSPAN18 and Myc-Orai1 using anti-Flag antibody (left) or anti-Myc antibody (right) in HEK-293T cells. (c) Co-IP analysis of interaction between Flag-TSPAN18 and Myc-Orai1 using anti-Flag antibody in Flag-TSPAN18 overexpressing DU145 cells.

**Supplemental Figure 2** The Western Blot analysis of TSPAN18 and STIM1 protein level in DU145 and PC-3 cells treated as indicated. The values are expressed as the mean ± s.d. of three independent experiments. ***p*<0.01, ****p* < 0.001, ANOVA with post hoc test or Student’s t test.


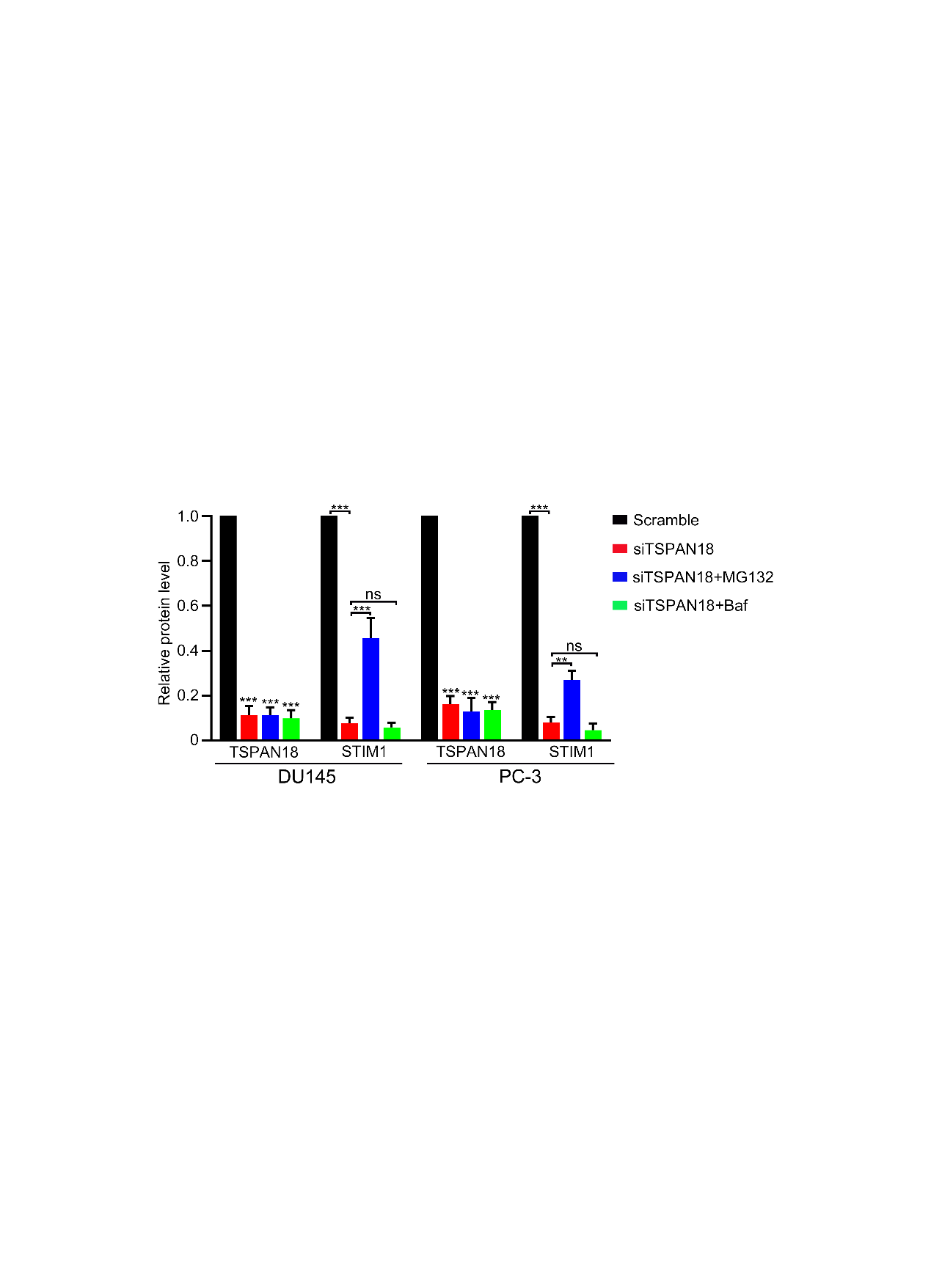

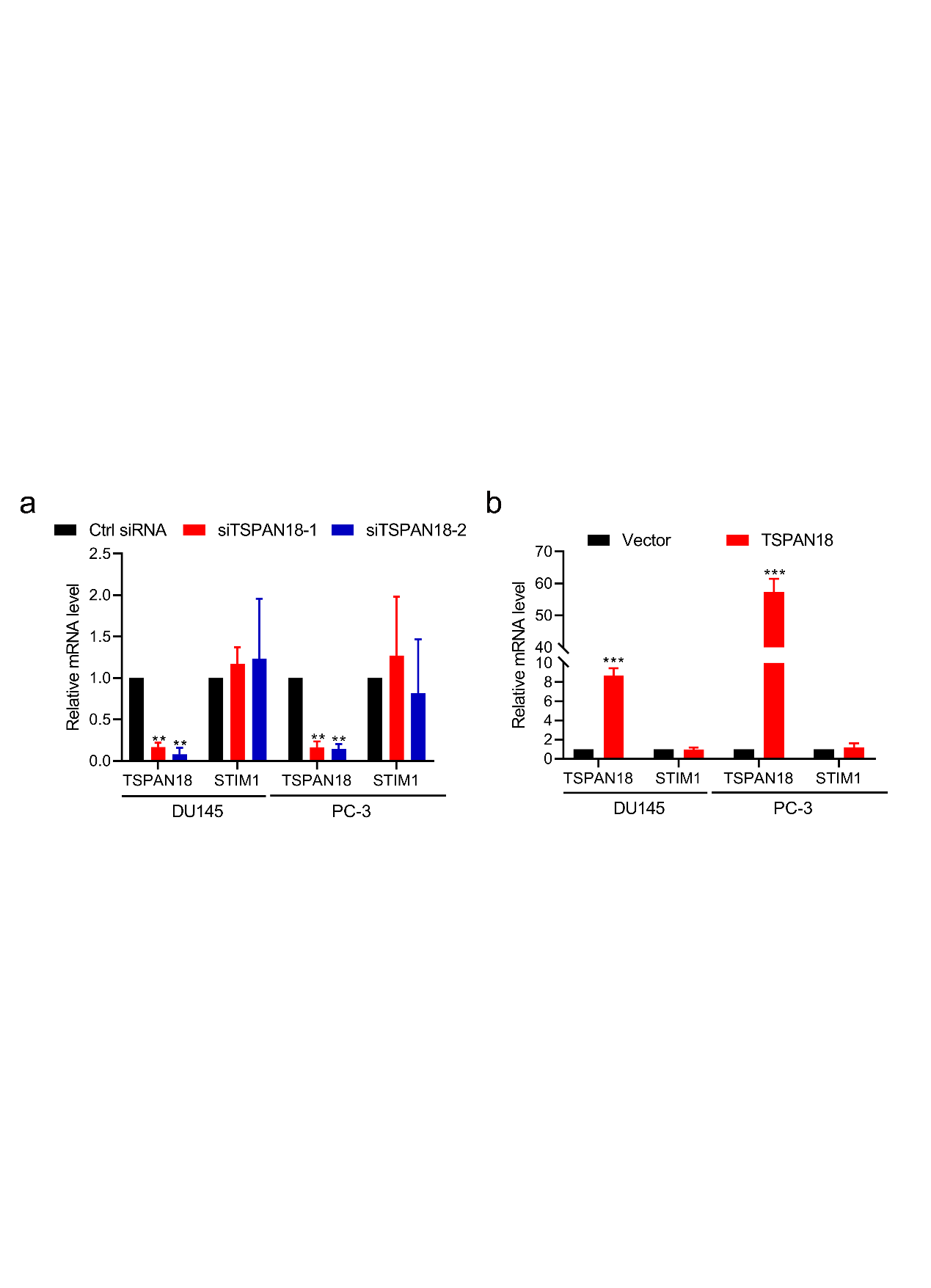
**Supplemental Figure 3** The qRT-PCR analysis of TSPAN18 and STIM1 mRNA level in DU145 and PC-3 cells treated as indicated. The values are expressed as the mean ± s.d. of three independent experiments. ***p*<0.01, ****p* < 0.001, ANOVA with post hoc test or Student’s *t* test.

**Supplemental Figure 4** The Western Blot analysis of TSPAN18 and STIM1 protein level in DU145 and PC-3 cells treated as indicated. The values are expressed as the mean ± s.d. of three independent experiments. ***p*<0.01, ****p* < 0.001, ANOVA with post hoc test. Baf: Bafilomycin, ns: no significance.


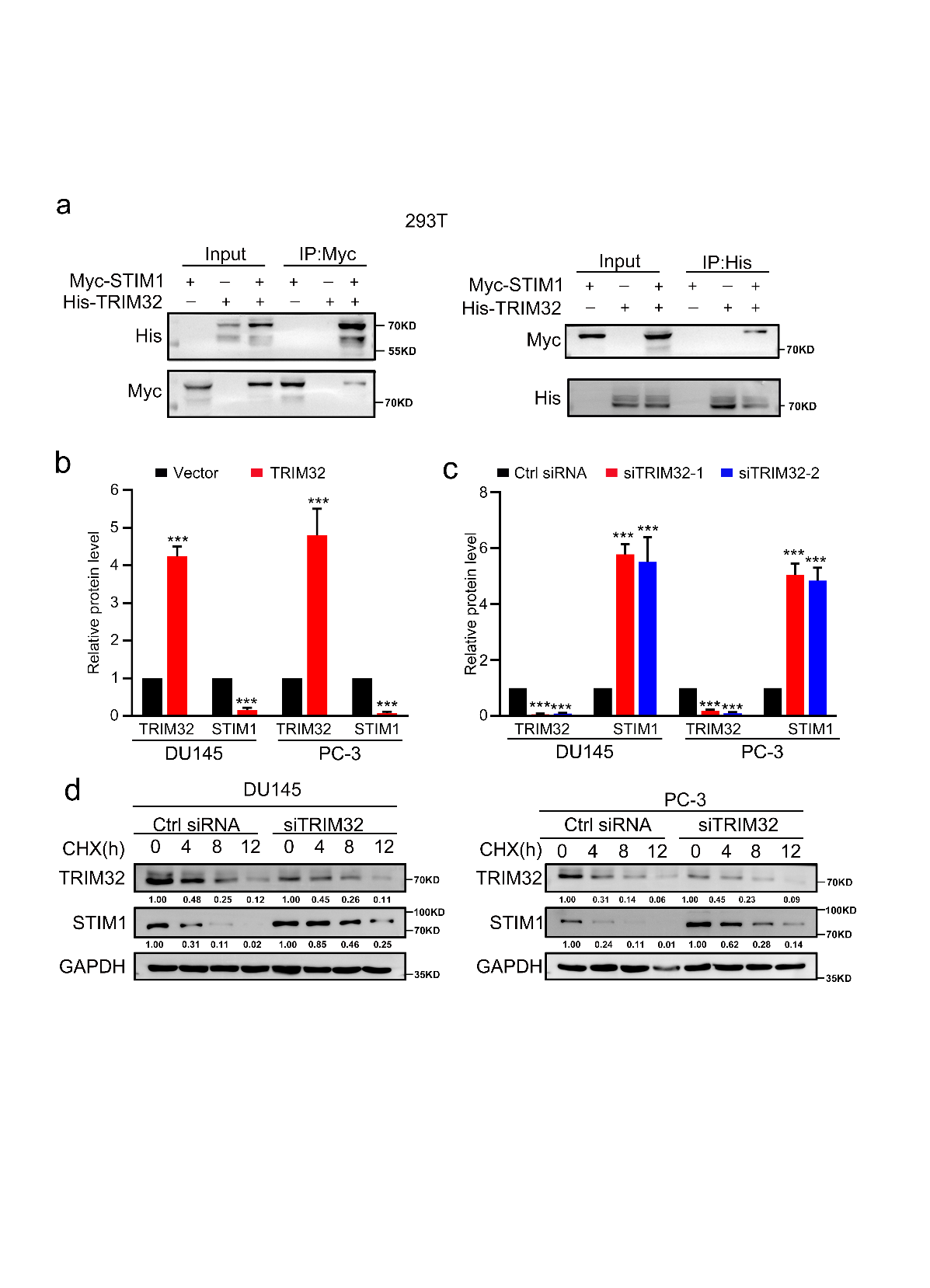

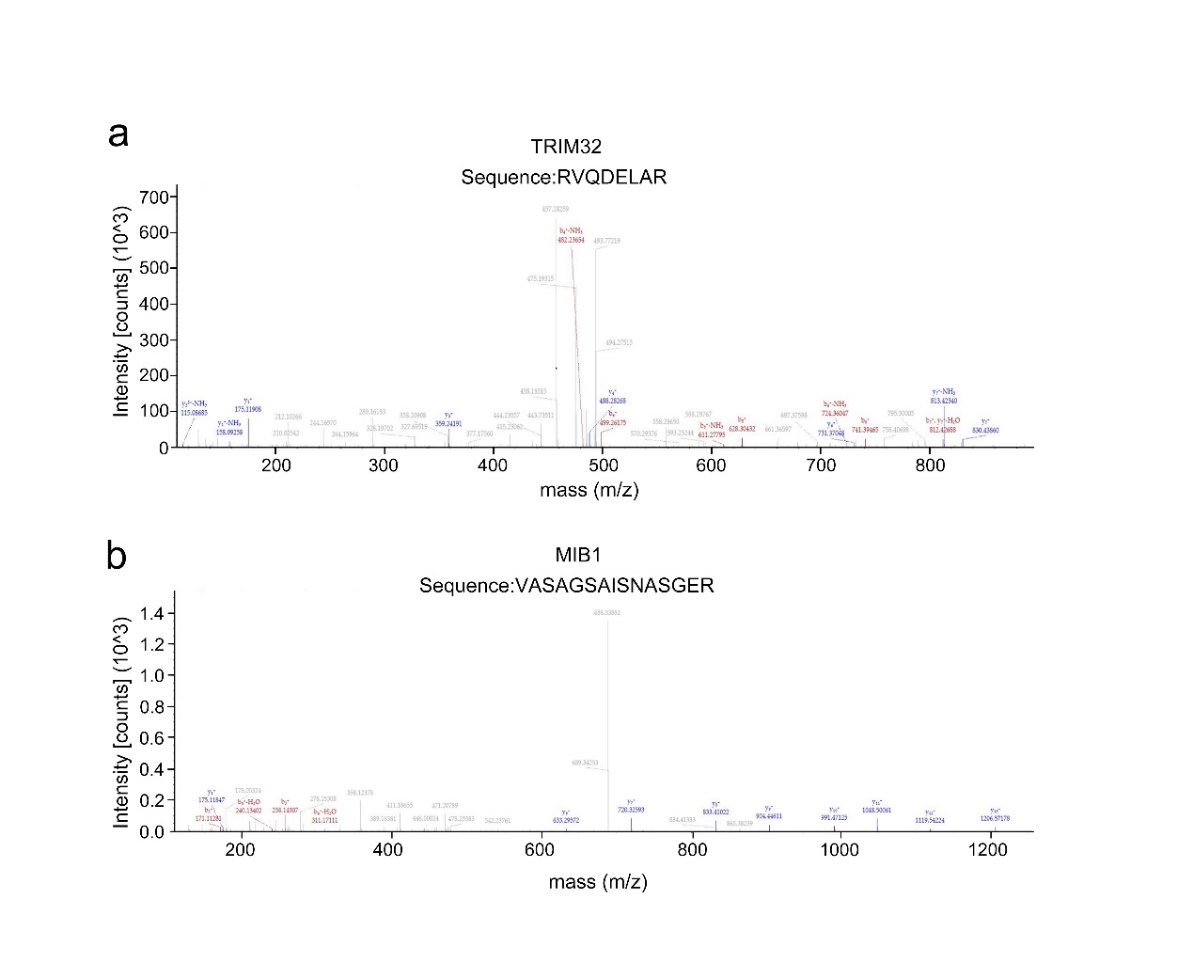
**Supplemental Figure 5** The representative peptide of TRIM32 (a) or MIB1 (b) from mass spectrometry.

**Supplemental Figure 6** (a) Co-IP analysis of interaction between exogenous STIM1 and


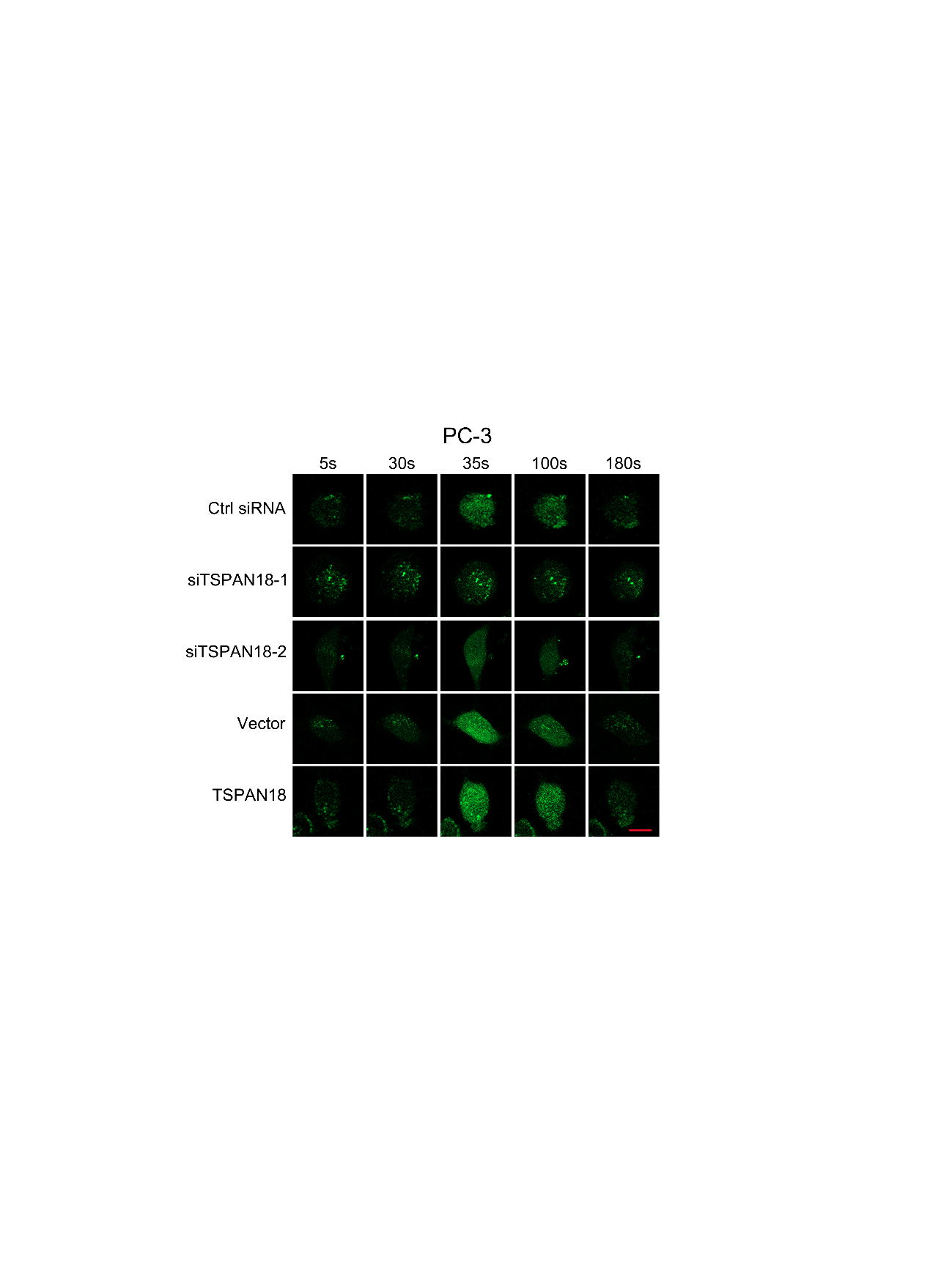
exogenous TRIM32 in HEK-293T cells transfected with His-TRIM32 plasmid and Myc-STIM1 plasmid using anti-Myc antibody(left) or anti-His antibody (right). (b-c) The Western Blot analysis of TRIM32 and STIM1 protein level in DU145 and PC-3 cells treated as indicated. The values are expressed as the mean ± s.d. of three independent experiments. ****p* < 0.001, Student’s t test or ANOVA with post hoc test. (d) The protein level of STIM1 in DU145 and PC-3 cells transfected with scramble or si-TRIM32 were monitored by WB at indicated times after cycloheximide (CHX, 20μg/mL).


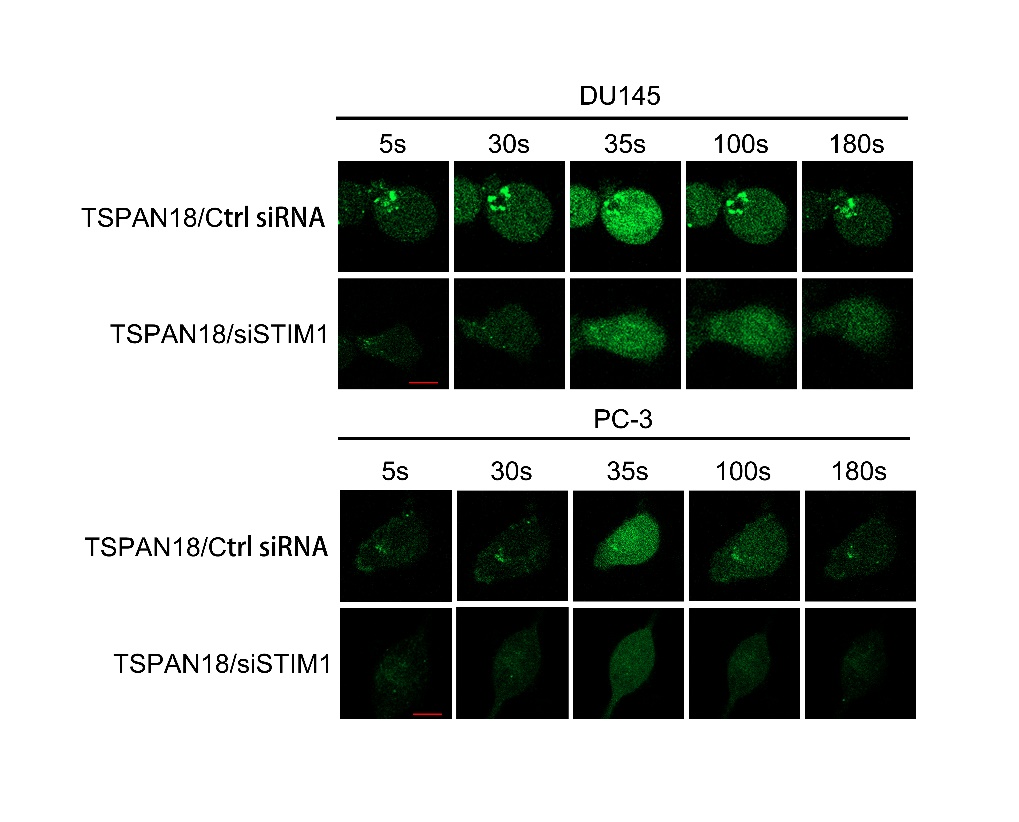
**Supplemental Figure 7** The representative time-lapse images of cytosolic Ca^2+^ level within PC-3 cells transfected with indicated siRNAs or plasmids. Scale bars: red, 50 μm.

**Supplemental Figure 8** The representative time-lapse images of cytosolic Ca^2+^ level within

indicated cells. Scale bars: red, 50 μm.


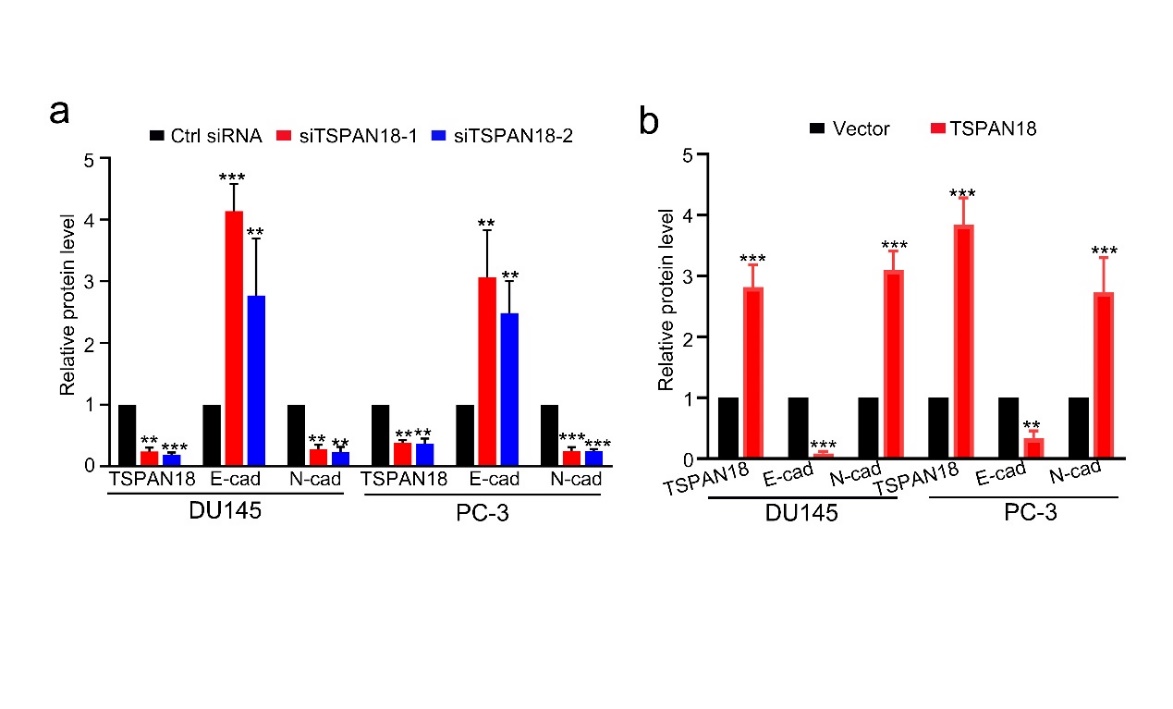

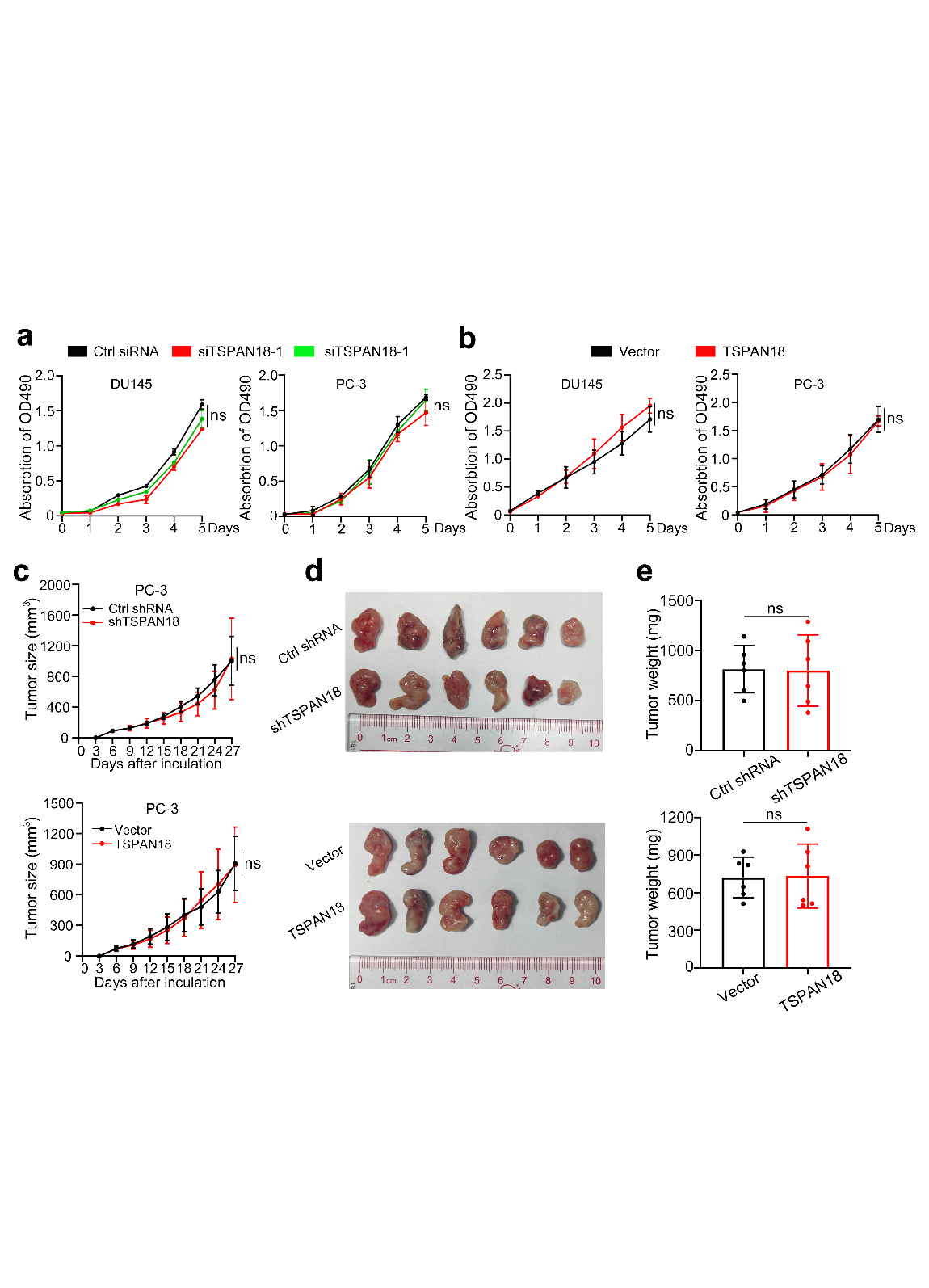
**Supplemental Figure 9** (a-b) Cell viability was evaluated in TSPAN18 knockdown or overexpressing DU145 and PC-3 cells. (c) Tumor growth curves are summarized in the line chart. The average tumor volume is expressed as the mean ± SD of six mice. (d) Representative images of the tumors of TSPAN18 knockdown or overexpression groups and their respective controls. (e) Tumor weights were measured after the tumors were surgically dissected. ns: no significance.

**Supplemental Figure 10** The Western Blot analysis of E-cadherin and N-cadherin protein level in DU145 and PC-3 cells treated as indicated. The values are expressed as the mean ± s.d. of three independent experiments. ***p*<0.01, ****p* < 0.001, ANOVA with post hoc test or

Student’s *t* test.


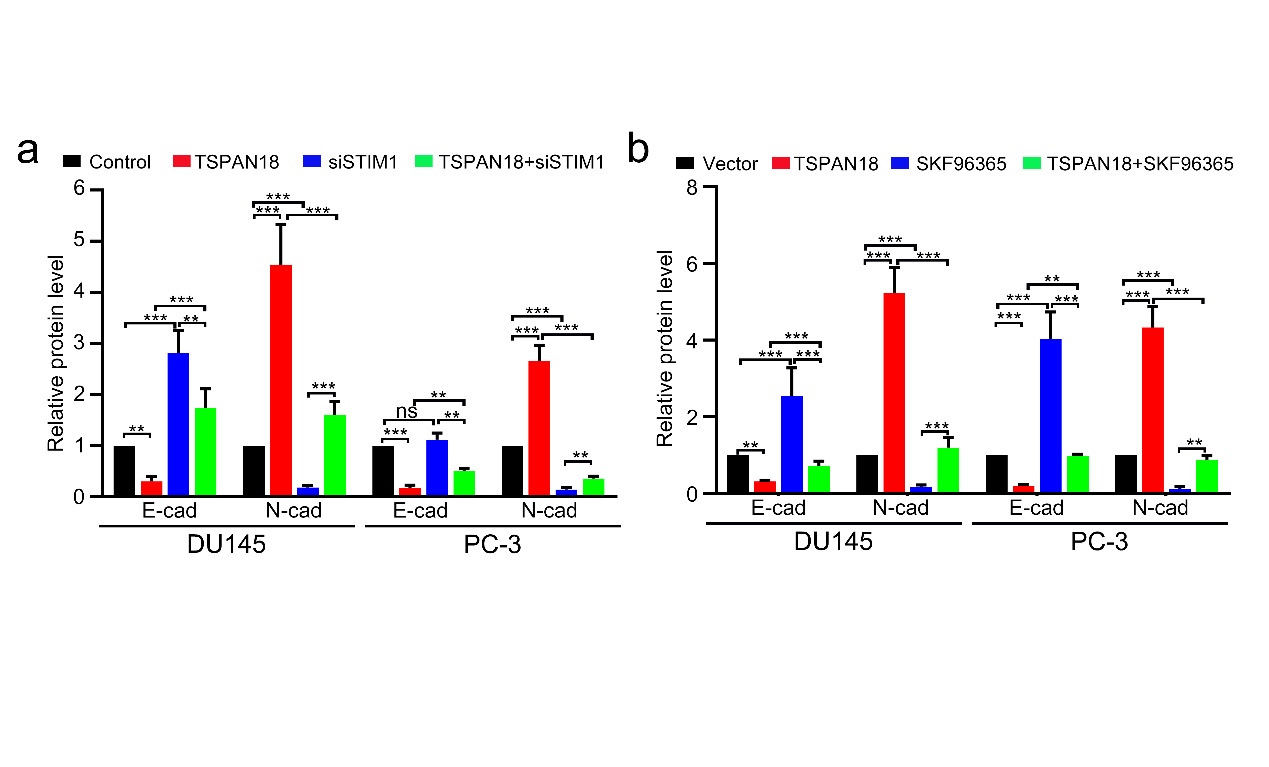

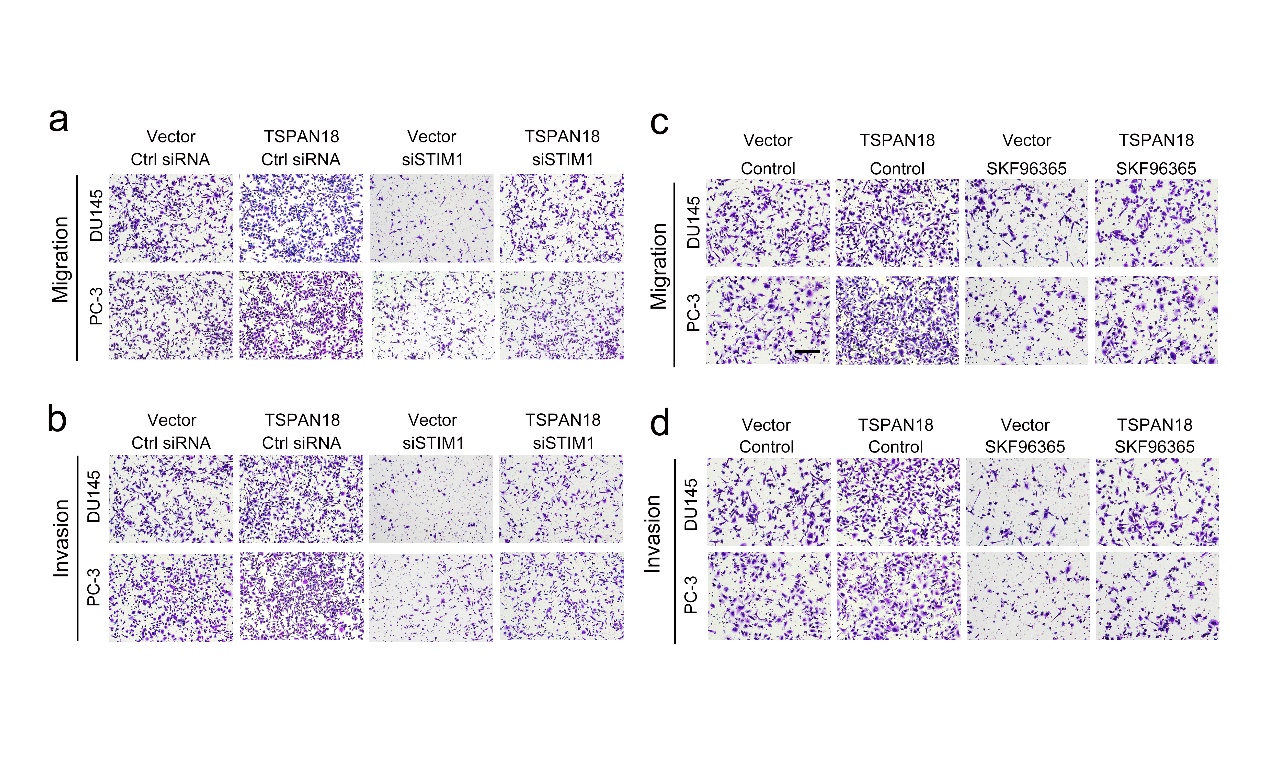
**Supplemental Figure 11** The representative images of migration and invasion assays using TSPAN18-overexpressing or control DU145 and PC-3 cells transfected with indicated siRNAs (a, b) or treated with SKF96365 or DMSO (c, d).

**Supplemental Figure 12** The Western Blot analysis of N-cadherin and E-cadherin protein level in DU145 and PC-3 cells treated as indicated. The values are expressed as the mean ± s.d. of three independent experiments. ***p*<0.01, ****p* < 0.001, ANOVA with post hoc test. ns: no significance.


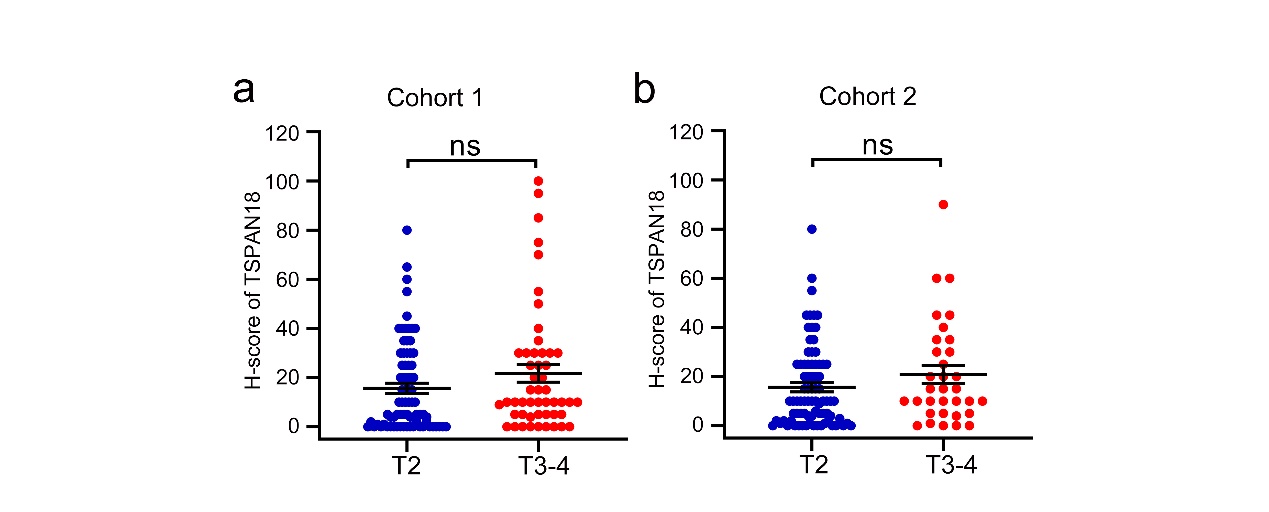


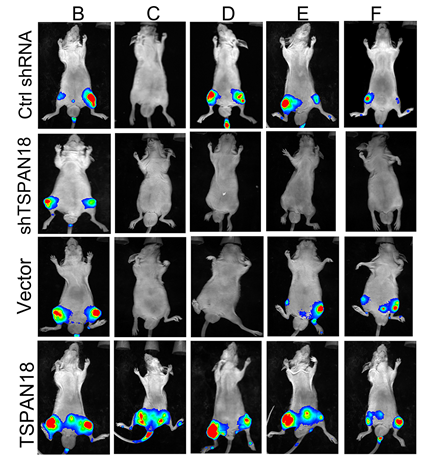


**Supplemental Figure 13** The representative bioluminescence images of the mice after 6 weeks of inoculations with indicated PC-3 cells.

**Supplemental Figure 14** The expression difference of TSPAN18 between low T stage PCa tissues and high T stage PCa tissues in Cohort 1 (a) and Cohort 2 (b). ns: no significance. Student’s *t* test.
